# Numerical investigation of ultimate capacity enhancement in corrugated web steel beams with different stiffening configurations

**DOI:** 10.1038/s41598-025-30614-0

**Published:** 2025-12-29

**Authors:** Aya Fouda, Mohamed Dabaon, Saad Eldeen Abdrabou, Mohamed Ghannam

**Affiliations:** 1 Structural Engineering Department, Faculty of Engineering, Mansoura University, Mansoura, 35516 Egypt; 2 Structural Engineering Department, Faculty of Engineering, Horus University-Egypt, New Damietta, 34517 Egypt; 3 Structural Engineering Department, Faculty of Engineering, Tanta University, Tanta, 31733 Egypt

**Keywords:** Strengthening, Shear strength, Load-carrying capacity, Corrugated web, Shear buckling mechanism, Steel, Engineering, Materials science

## Abstract

Corrugated web steel beams (CWSBs) are widely applied in modern construction due to their high strength-to-weight ratio and enhanced shear stability. However, research on strengthening strategies to further improve their structural efficiency remains limited. This study addresses the existing research gap by presenting a validated finite element investigation of corrugated web steel beams (CWSBs) with different stiffening configurations. Five stiffening configurations (horizontal stiffeners, vertical doubler plates, inclined stiffeners, diagonal doubler plates, and bracing stiffeners) were examined under uniformly distributed loads to evaluate their effectiveness in enhancing load-carrying capacity and shear performance. Numerical models were developed and validated against previously published experimental and numerical data, demonstrating high accuracy. A parametric study was conducted to assess the influence of stiffener type, configuration, and inclination angle on the performance of CWSBs. Results reveal that horizontal stiffeners placed at one-fifth of the web height outperform mid-height placement in enhancing ultimate capacity. Inclined stiffeners achieve optimal performance at 45°, aligned with the principal stress direction. Bracing stiffeners provide the greatest overall enhancement by stabilizing multiple web folds and efficiently redistributing shear forces. Among all configurations, the 45° angle bracing delivered the highest strength-to-weight ratio of 5.52 ton/kg. These findings offer actionable insights for optimizing the performance of CWSBs in applications where both strength and material efficiency are critical.

## Introduction

Corrugated web steel beams (CWSBs) are widely used in civil engineering, particularly in industrial buildings and bridges. Introduced in Europe during the 1960s and applied in bridges by the 1980s^1^. Various corrugation shapes have been developed, including rectangular, triangular, sinusoidal, and the widely used trapezoidal shape^2,3^. CWSBs offer better performance than conventional welded plate girders by enhancing stability, load capacity, and out-of-plane stiffness and load capacity while delaying shear buckling without requiring vertical stiffeners. CWSBs are lightweight and cost-effective due to thinner web plates that enhance fatigue life^1,4^. Studies show flat web beams need over twice the steel volume as corrugated webs for the same shear capacity^2^. The unique configuration of the corrugated web results to the “accordion effect”^4^, meaning that the flanges carry the entire bending moment, whereas the corrugated web purely carries only shear forces, as defined by Eurocode 1993-1-5.This separation simplifies analysis by eliminating shear–moment interaction. Corrugated webs can fail due to local, global, and interactive shear buckling and material yielding^5–7^. Buckling occurred before yielding, except in stocky webs^8–10^.

Several studies have examined behaviors of CWSBs, such as shear^11–13^, flexural^8,14–16^, and lateral torsional behavior^6,7,17,18^. Other studies showed that flange constraints significantly affect post-buckling strength and elastic shear stiffness^6,19^. Initial geometric imperfections affect CWSBs strength predictions. FEM studies showed that hw/200 imperfections reduced strength by 25%. Thus, using a maximum fold length/200 or web thickness as imperfection amplitude is recommended, with the first mode being the most critical^9,19^. The shear strength of CWSBs is also influenced by strain hardening and post-buckling behavior. Amani et al.^19^ reported an 8–18% shear strength increase due to strain hardening, while Wang et al.^20^ stated that elastoplastic buckling and initial imperfections reduced shear strength. Leblouba et al.^10^ and Zhang et al.^21^ found that 50–60% of shear strength retained post-buckling. ﻿Alikhanifard et al.^28^ observed uniform shear stress before buckling, with tensile stresses dominating post-buckling resistance.

Analytical formulas were developed for predicting shear strength of (CWSBs). Leblouba et al.^22^ found the design shear strength in EN 1993-1-5 to be conservative and proposed a simplified model for improved accuracy^23^. Driver et al.^9^ observed strength overestimation caused by simply supported edge assumptions and geometric imperfections. Sause and Braxtan^24^ proposed a new formula including imperfections and residual stresses. Wu et al.^25^ validated design model of Leblouba et al.^23^ and proposed modifications for rigid juncture between flanges and web. Wang et al.^5,11,12^ refined buckling stresses formulas, and modified the shear strength design to account for buckling interactions. Leblouba et al.^26^ aligned shear design guidelines for (CWSBs) with standards AISC 360 and CSA-S16, recommending optimized resistance factors. Hassanein et al.^27^ and Alikhanifard et al.^28^ developed a new interactive shear buckling strength formula and recommended the Sause and Braxtan’s formula. Shrif et al.^29^ created a model to predict shear strength better than existing models. Mohamed et al.^30^ developed a design equation for high-strength steel (CWSBs) using deep learning. Wang et al.^11,31^ and He et al.^32^ emphasized the need for revised calculations for steel I-girders with stiffened corrugated webs.

Parametric studies have been essential in identifying optimum configurations for (CWSBs), to improve shear performance^21,28,33,34^. Hassanein et al.^33^ found that corrugation angles over 30° help prevent global buckling. Shrif et al.^29^ determined that (35°–45°) corrugation angles maximize shear strength. Zhong et al.^35^ also indicate that CW beams with trapezoidal, zigzag, and sinusoidal profiles exhibit similar collapse behavior, whereas rectangular CWs show lower bearing capacity and are not recommended for structural applications. Other key parameters include web depth, thickness which are the most influential parameters. Mohamed et al.^30^ recommended a web height-to-width ratio of 4.0 for effective shear design in HSS corrugated web. Wang et al.^11^ stated how web thickness and height affect buckling modes. Additionally, studies explored how various parameters influence the shear buckling coefficient, emphasizing its dependence on panel dimension and boundary conditions^6,10,36,37^.

Recently, most research has focused on enhancing the performance of CWSBs through various approaches. The use of high-strength steel in beam’s web demonstrates higher shear and buckling capacities compared to normal-strength steel^28^. Additionally, partially corrugated webs have shown improved shear strength and deformation capacity over conventional webs^38^. While the integration of horizontal stiffeners has been found to improve local axial stiffness and overall shear strength^31,32^. Moreover, innovative strengthening techniques such as fiber reinforced polymer (FRP) were utilized. This application led to an enhancement in web stiffness and a reduction in critical stresses^39^. Moreover, recent studies by Zhong et al.^35^ have proposed structural reinforcement strategies for CW beams with web openings, demonstrating significant improvements in load-carrying capacity and collapse resistance. Despite these advancements, the effects of various stiffener configurations and web reinforcement strategies on the overall performance of CWSBs, particularly their influence on shear stress distribution and ultimate load remain insufficiently investigated. This gap motivates the present study, which aims to conduct a systematic parametric investigation using numerical modeling to evaluate the performance of CW beams without web openings under different stiffening strategies.

## Research objectives

Based on the aforementioned, the focus of this study has been on the shear behavior of CWSBs, using experimental, finite element analyses and design equations. Furthermore, the use of high-strength steel, partially corrugated webs (PCWs) and the addition of horizontal stiffeners have been explored. Thus, the main objectives of this study are as follows.

- Evaluate the effect various stiffener configuration on the performance and load-capacity of CWSBs using nonlinear finite element analysis.
- Determine the shear stress enhancement of CWSBs with stiffener subjected to uniform distributed load.
- Propose effective profiles of stiffener configurations for reducing shear stress and load-capacity of CWSBs.
- Determine the profile of stiffener that provide the best enhancement to CWSBs.

## Research methodology

**Fig. 1:**
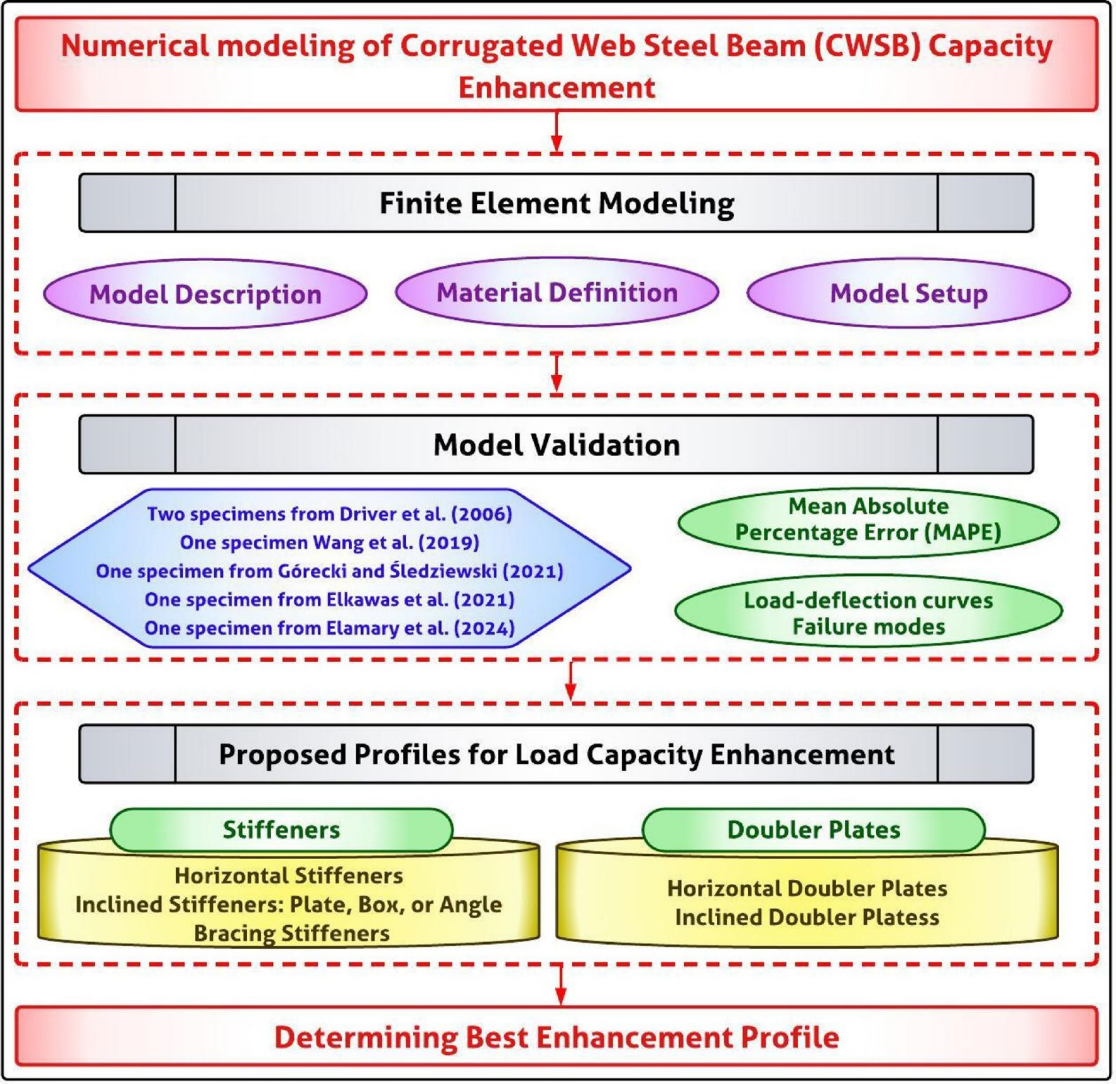
Methodological flow chart adopted in the study.

The previously stated objectives can be achieved according to the following methodology: (1) Constructing a FE model to simulate the behavior of corrugated web steel beams with stiffener subjected to subjected to uniform distributed load. (2) Validating and Calibrating the proposed FE model against previous work of corrugated web beams experimental and FE results, and (3) Using the proposed FE model to conduct a full parametric study to investigate the effect of different stiffener configuration, location, inclination angles, and the placement on the capacity and shear stress of corrugated web steel beams with stiffener as illustrated in the Fig. 1.

### Numerical modeling

Finite element analysis (FEA) has become an essential tool in analyzing the structural behavior of complicated systems, and one of the leading software programs used for this purpose is ABAQUS. The finite element (FE) method is widely used for numerical problem-solving, as it models a physical structure as an assembly of individual elements interconnected at particular nodes which is modeled using algebraic relationships to provide an idealized depiction of actual behavior^36^. This approach has often been preferred over full-scale experimental techniques in the examining the behavior of corrugated web beams to minimize the manufacturing expenses. In this study, (FEA) is performed by using ABAQUS software. Driver et al.^9^ experimental data is used in the present research for validation purposes. To further proof the reliability of the proposed FE results, the experimental tests of Wang et al.^31^ and Elkawas et al.^17^ and Górecki and Śledziewski^40^ are also simulated. The methods used to construct the model and the procedure for determining the capacity and shear stress of steel beams with stiffened corrugated webs under uniform load are descried by detail in the following sections.

#### Material modelling and section properties

The steel material behavior was represented using true stress–plastic strain curves, which were obtained by converting the engineering stress–strain curves into true stress–plastic strain curves based on Eqs. (1–3)^41^. The true stress–plastic strain curve has been of steel (HPS-485 W) has been assumed to be used in this study^42^. The material properties were the data of tension test on steel performed by Caroline et al.^43^. Elastic modulus E has been evaluated to be 200 GPa, Poisson’s ratio ν equals 0.3 and the yield stress of steel ${F}_{y}$ was assumed as 490 MPa. Thicknesses of shell were assigned using the Property function in ABAQUS.

1 $${\delta }_{\text{true }}={\delta }_{eng}\left(1+{\varepsilon }_{eng}\right)$$

2 $${\varepsilon }_{true}=ln\left(1+{\varepsilon }_{eng}\right)$$

3 $${\varepsilon }_{\text{true }}^{pl}=\mathrm{ln}\left(1+{\varepsilon }_{eng}\right)-\frac{{\delta }_{\text{true }}}{{E}_{0}}$$

#### Finite element type and mesh

All elements of the beam (flange, web and stiffener) were modelled using Shell geometries. “Shells” are structures characterized by one dimension being considerably smaller than the other dimensions^36^. The flange and stiffener were constructed using a planar shell while web was constructed using an extrusion shell. Four-node shell elements with reduced integration (S4R) were utilized to model corrugated web beams in the current analysis, following the approach of previous studies^44,45^. The S4R element, a type of thin shell element which is appropriate for modeling structures where the thickness is less than one-fifteenth of the characteristic length. “S” represents a conventional stress/displacement shell, “4” indicates the number of nodes, and “R” refers to reduced integration. S4R elements have four integration nodes with linear base function. Each node of the element has six degrees of freedom, including three translational and three rotational degrees of freedom.

**Fig. 2:**
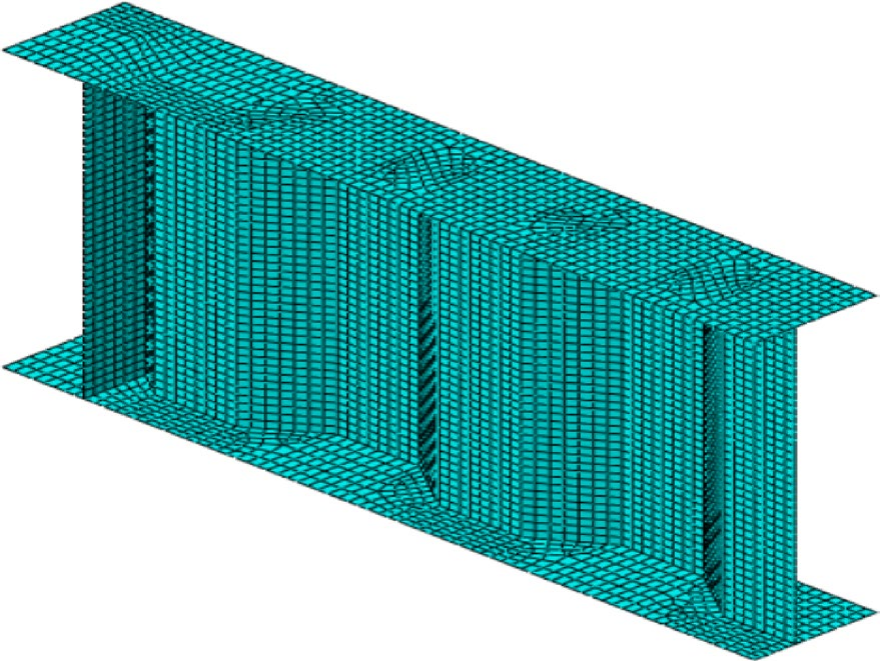
Structural FE meshing of a typical corrugated web beam.

**Fig. 3:**
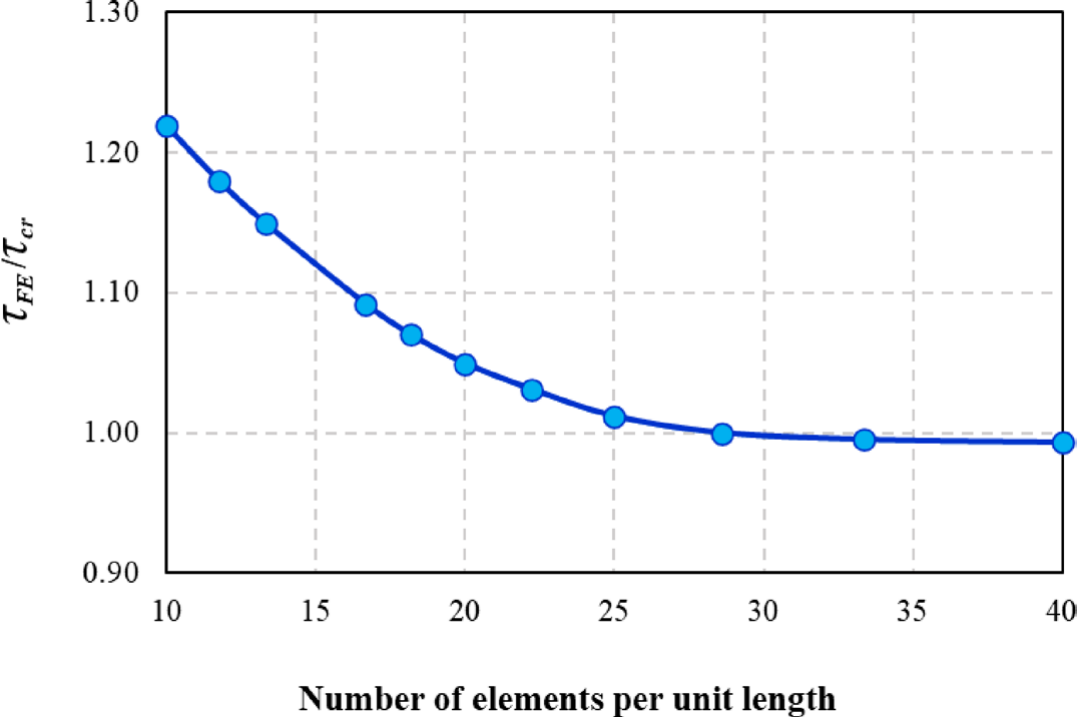
Results from mesh convergence study.

A properly constructed finite element mesh is essential for achieving accurate results. The study compared two meshing approaches: a merged instance mesh and a master–slave mesh, where the flange and web were meshed separately. Results indicated that no significant differences in the results obtained^36^, so meshing techniques was merging web and flange part instances to form a single component and applying a free mesh using the Advancing Front algorithm with mapped meshing. Figure 2 shows the element size of the web, stiffeners and flanges was selected 35 × 35 mm which satisfies the condition of (*h*_*w*_/20) and (1.0 *t*_*f*_) based on member size^30,36^ and a convergence study which indicated that mesh size of 35 mm was considered as the best size for achieving accurate results and save the computation, as illustrated in Fig. 3.

#### Boundary conditions and loading

**Fig. 4:**
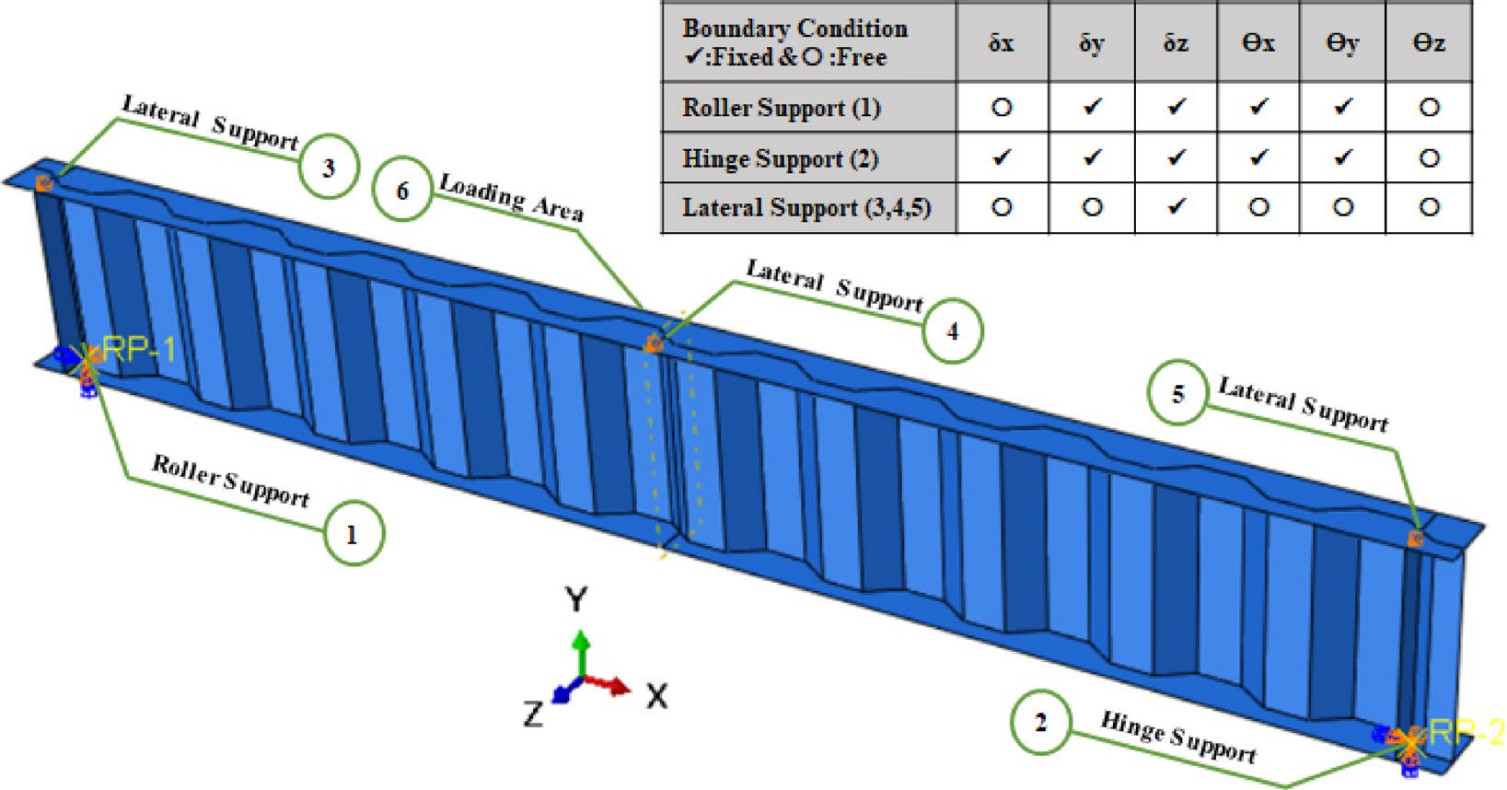
Loading and boundary condition of numerical models.

The used boundary conditions for the current finite element model in linear buckling and nonlinear analyses were hinge support on one side of the beam where the restrained displacement were in x, y, z-direction and rotational degree of freedom at (x, y axis) and roller support on the other side where the restrained displacement were y, z-direction and rotational degree of freedom at (x, y axis).Vertical Stiffener in the model have been located at support reaction locations to prevent local crippling of the flange where the web was welded. To prevent beams from failure by Lateral torsional buckling, the FE models were laterally restrained at the points C, D and E, as shown in Fig. 4. Distributed uniform Load have been statically applied along the upper flange of beam. The flange-web boundary is achieved using a sufficiently thick flange, with a flange-to-web thickness ratio greater than 5 to prevent local buckling of the flange^46^.

#### Modelling of equivalent geometrical imperfections

To obtain an accurate numerical analysis and real results, EN1993-1-5^47^ states that geometric imperfections should be added to the FE models before conducting the nonlinear numerical analysis. The imperfection amplitude was equal to the thickness of the corrugated steel web^9,22,27^. A two-step method is used to model a corrugated web beam having equivalent imperfections. First, a linear elastic buckling analysis is performed using the buckle step in ABAQUS to generate the expected buckling modes and nodal displacements. In the second step, geometrical imperfections are applied to the model by adding the first positive elastic buckling mode, with a scaling factor equal to the equivalent imperfection amplitude.

#### Interaction between different elements

The ABAQUS model allowed easy assembly of the corrugated web, top flange and bottom flange using translation and rotation functions to arrange individual elements into correct positions. The completed model is shown in Fig. 4. Tie constraints effectively connect different components in an Abaqus FEA model, ensuring they behave as a cohesive structure during simulation, regardless of mesh or material differences. The vertical stiffeners were tie constraints in the middle of inclined fold at end supports. The interactions between steel corrugated web beam and different configuration stiffeners are implemented using tie constraints to represent welding between them.

### Model validation

To validate the accuracy of the suggested FE models, numerical simulations were conducted using ABAQUS program for several experimental tests on the behavior of corrugated web steel beams. These tests include specimens from various studies as follows: Driver et al.^9^, Wang et al.^31^, Górecki and Śledziewski^40^, Elamary et al.^48^, and Elkawas et al.^17^. An overview of these validations is mentioned in the following section. Driver et al.^9^ investigated the shear behavior of corrugated web girders and the effect of web initial geometric imperfections using two full-scale specimens to verified the Current FE model. Wang et al.^31^ studied shear performance of stiffened corrugated web on four steel I-beam with corrugated web considering various stiffener arrangement, among test specimens there was a specimen without stiffener which has been employed in the current study for validation. While Elamary et al.^48^ analyzed effect of the inclined folds welded or non-welded between the web and flanges on five full-scale CWBs, among one specimen was fabricated with continuous welding between the corrugated web and flanges which has been used in the current study for comparison. Moreover, Górecki and Śledziewski^40^ analyzed the influence of the geometric parameters on the behavior of CWSBs using eight samples and Elkawas et al.^17^ conducted parametric studies on the behavior of three CWSBs, two of which were used to confirm further the reliability of the results of the suggested FE model.

#### Material properties

Table 1 presents the mechanical properties where yield stress (Fy) and ultimate stresses (Fu). Young’s modulus of elasticity $E$ and Poisson’s ratio $\nu$ for the corrugated web and flange, also provides the load positions applied during testing and initial imperfection measured of CWSBs tested. The initial buckling mode was set as the defect shape before conducting the nonlinear finite element (FE). The amplitude of this imperfect shape was incorporated into the FE model in ABAQUS, utilizing the initial imperfection measured for these beams in experimental tests. Which was approximately equal to the thickness of the corrugated steel web or the span divided by 1000. The material behavior for validated models was represented using true stress–plastic strain curves and anther details are accessible in^9,17,31,40,48^.

**Table 1:** Beam characteristics of experimental tests simulated by FE models.

Beams	Mechanical properties						Load position	Imperfection $\frac{L}{1000}\; or \; {t}_{w}$
	Web		Flange					
	${F}_{y}$ (MPa)	${F}_{u}$ (MPa)	${F}_{y}$ (MPa)	${F}_{u}$ (MPa)	E (GPa)	ν		
G7A^9^	465	–	499	–	200	0.3	Point load applied 1 m from midspan	4.06
G8A^9^	465	–	499	–	200	0.3		5.72
BS200/3^40^	415	660	415	660	197.8	0.3	Two-point load placed 636 mm from each end	2.5
W1^31^	400	524	427	546	203	0.3	Point load at midspan	3
G-400-100^17^	304	371	295	462	200	0.3	Two-point load at the 600 mm from each end	2
CW35IFWL^48^	420	530	225	320	200	0.3	Line load at midspan	2

#### Geometric parameters

Details of the geometrical dimensions of the tested beams are provided in Table 2, including beam dimensions where flange width $$({b}_{f})$$, flange thickness $$({t}_{f})$$, web height $$({h}_{w})$$, and web thickness $${(t}_{w})$$ and also, corrugation geometry where the unit wavelength of corrugated web $$\left(s\right)$$, horizontal length of one corrugation ($$q)$$, parallel fold $$\left(b\right)$$, inclined fold width $$\left(c\right)$$, and the projected width of inclined fold $$(d)$$. The corrugation depth ($$hr$$) and angle. Span of simple beam (S) which were modelled to 10 full corrugation waves with total length (L).

**Table 2:** Geometrical dimensions of experimental tests simulated by FE models.

Beams	Corrugation Geometry							Beam dimensions					
	b	D	c	${h}_{r}$	a	q	$\alpha$	${b}_{f}$	${t}_{f}$	${h}_{w}$	${t}_{w}$	S	L
G7A^9^	300	200	250	150	–	1000		450	50	1500	6.3	11,000	11,600
G8A^9^	300	200	250	150	–	1000		450	50	1500	6.27	11,000	11,600
BS200/3^40^	–	–	–	–	55	200	–	180	8	250	3	2228	2500
W1^31^	110	55	105.5	90	–	400	30°	200	25	1200	3	4200	4400
G-400-100^17^	100	28.9	57.7	50	–	300	30°	100	16	400	3	3000	3100
CW35IFWL^48^	350	100	141.4	100	–	900	45°	200	8	400	3	1800	1900

#### Validation results

**Fig. 5:**
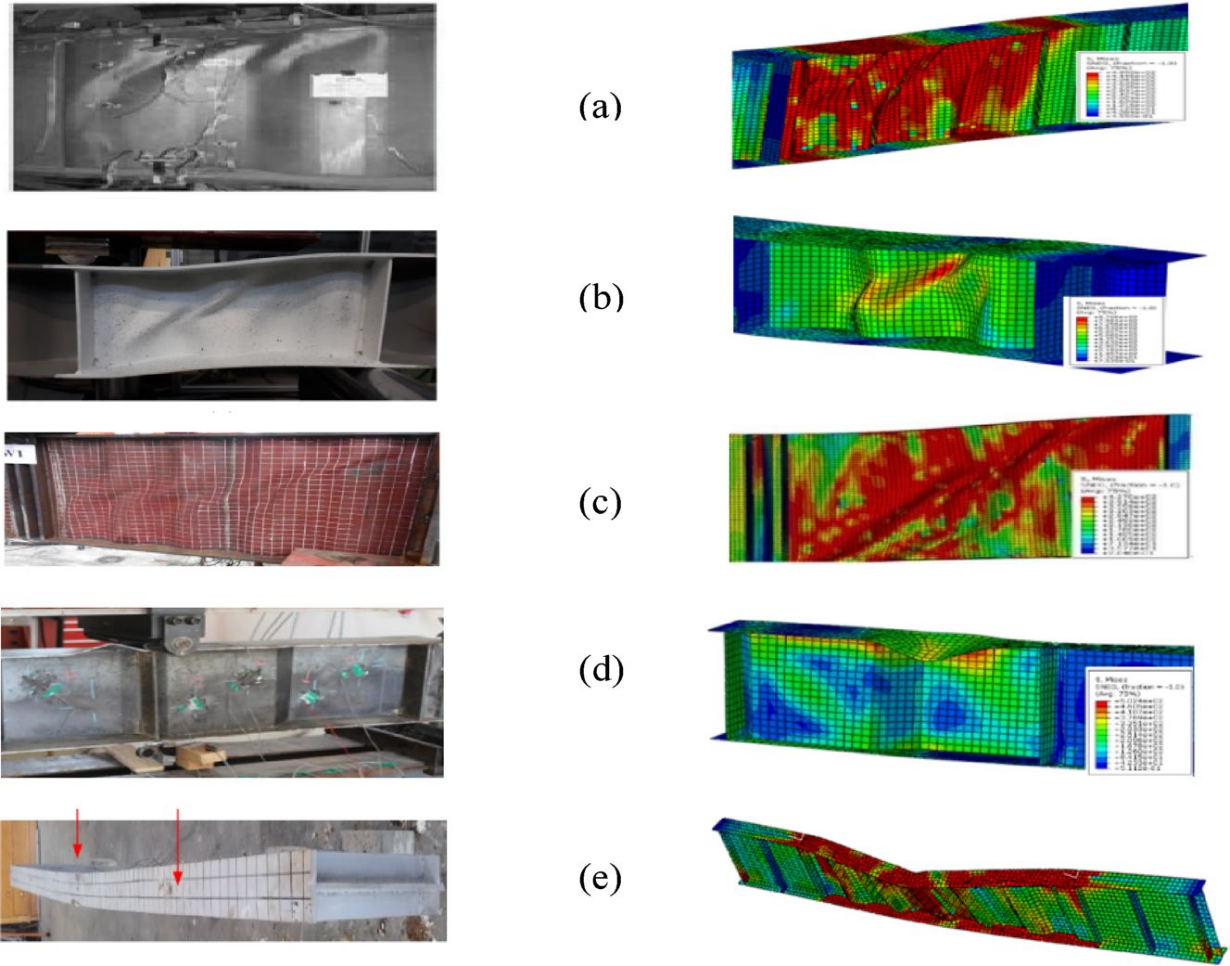
Final failure modes observed in the experimental and numerical tests (**a**) G7A^9^, (**b**) BS200/3^40^, (**c**) W1^31^, (**d**) CW35IFWL^48^, (**e**) G-400-100^17^.

The final failure modes observed in the experimental tests G7A by Driver et al.^9^ that the girders failed due to web buckling in the regions involving folds 2–4 near the support and FE model accurately simulated failure modes which are shown in Fig. 5a. The proposed finite element model for tested specimen w1 by Wang et al.^31^ effectively simulated failure mode, with the buckling range concentrated at the middle of the web height as illustrated in Fig. 5b. The FE model for Specimen G-400-100 by Elkawas et al.^17^ demonstrated the ability to reliably simulate the failure mechanism with accuracy, showing that this girder failed by lateral-torsion buckling as exposed in Fig. 5c without any distortion or local buckling in its cross-sectional elements. The proposed FE for tested beam CW35IFWL by Elamary et al.^48^ accurately simulated failure modes as illustrated in Fig. 5d, showing that the beam failed owing to local buckling of flange only close to the load line position. The FE model for Specimen BS200/3 by Górecki and Śledziewski^40^ confirmed the ability to reliably simulate the failure mechanism with accuracy, showing that the primary cause of damage was web instability as exposed in Fig. 5e, which started with local buckling in the straight sections between folds, leading to plastic deformation lines near the upper flanges.

**Fig. 6:**
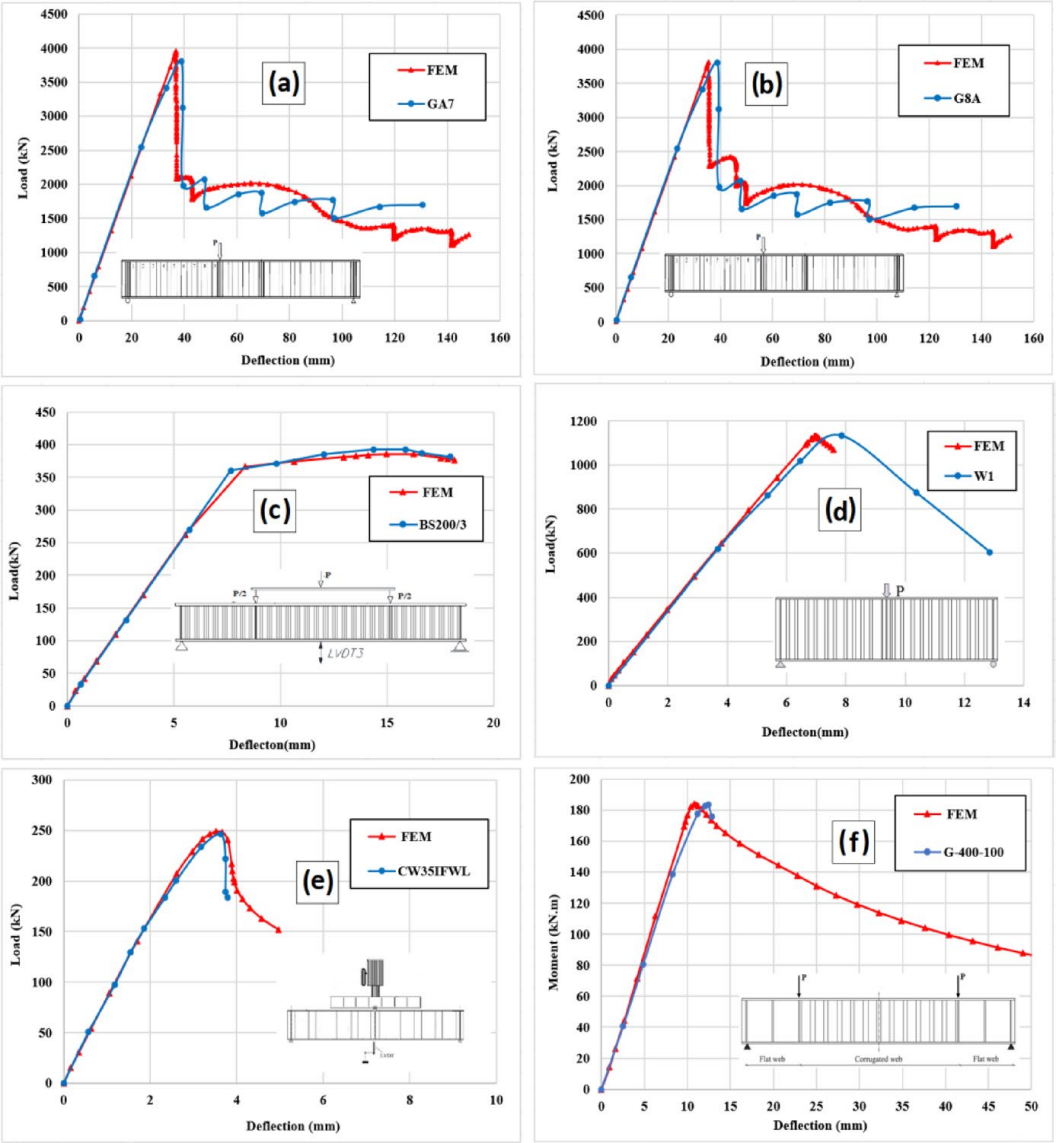
Comparison between experimental and finite element modeling of load vs. mid-span displacement curves (**a**) G7A^9^, (**b**) G8A^9^, (c) BS200/3^40^, (**d**) W1^31^, (**e**) CW35IFWL^48^, (**f**) G-400-100^17^.

Figure 6 indicated that the FE model accurately simulated the load versus midspan deflection for the verified beams. The load–midspan deflection curves of the FE models followed the same trend as the experimental specimens confirming sufficient accuracy of the finite element models. Table 3 compares the FE results (${\mathrm{P}}_{\mathrm{ul},\mathrm{FE}}$ and ${\updelta }_{\mathrm{ul},\mathrm{FE}}$) and experimental results (${\mathrm{P}}_{\mathrm{ul},\mathrm{EXP}}$ and ${\updelta }_{\mathrm{ul},\mathrm{EXP}}$) including the ultimate load and deflection. The mean absolute percentage error (MAPE) is the used metric to compare the results. Results showed that MAPE valued 1.19% and 4.80% for ultimate load and deflection, respectively. The MAPE values are acceptable in practice as recommended by previous research^17^.

**Table 3:** Comparison between numerical and experimental load carrying capacities and mid-span displacement.

Girder	P_ul,EXP_ (kN)	P_ul,FE_ (kN)	δ_ul,EXP_ (mm)	δ_ul,FE_ (mm)	P_ul,FE_/P_ul,EXP_	δ_ul,FE_/δ_ul,EXP_	$\left| {\frac{{{\mathrm{P}}_{{{\mathrm{ul,FE}}}} - {\mathrm{P}}_{{{\mathrm{ul,EXP}}}} }}{{{\mathrm{P}}_{{{\mathrm{ul,Exp}}}} }}} \right|$*100	$\left| {\frac{{{\updelta}_{{{\mathrm{ul,FE}}}} - {\updelta}_{{{\mathrm{ul,EXP}}}} }}{{{\updelta}_{{{\mathrm{ul,Exp}}}} }}} \right|$*100
G7A^9^	3882.5	3957.791	39.44	36.79	1.019	0.93	1.95%	6.72%
G8A^9^	3728.5	3808.57	36.23	35.40	1.021	0.98	2.15%	2.28%
BS200/3^40^	393.02	385.8	16.37	16.26	0.982	0.99	1.88%	0.67%
W1^31^	1134.6	1132.31	8.05	7.45	0.998	0.93	0.20%	7.47%
G-400-100^17^	612	613.63	12.45	11.26	1.003	0.91	0.27%	9.60%
CW35IFWL^48^	245	245.9	3.8	3.65	1.004	0.96	0.37%	1.57%
Average					1.005	0.968	–	–
Standard deviation					0.010	0.028	–	–
Mean Absolute percentage error (MAPE)							1.19%	4.80%

### Parametric study

**Fig. 7:**
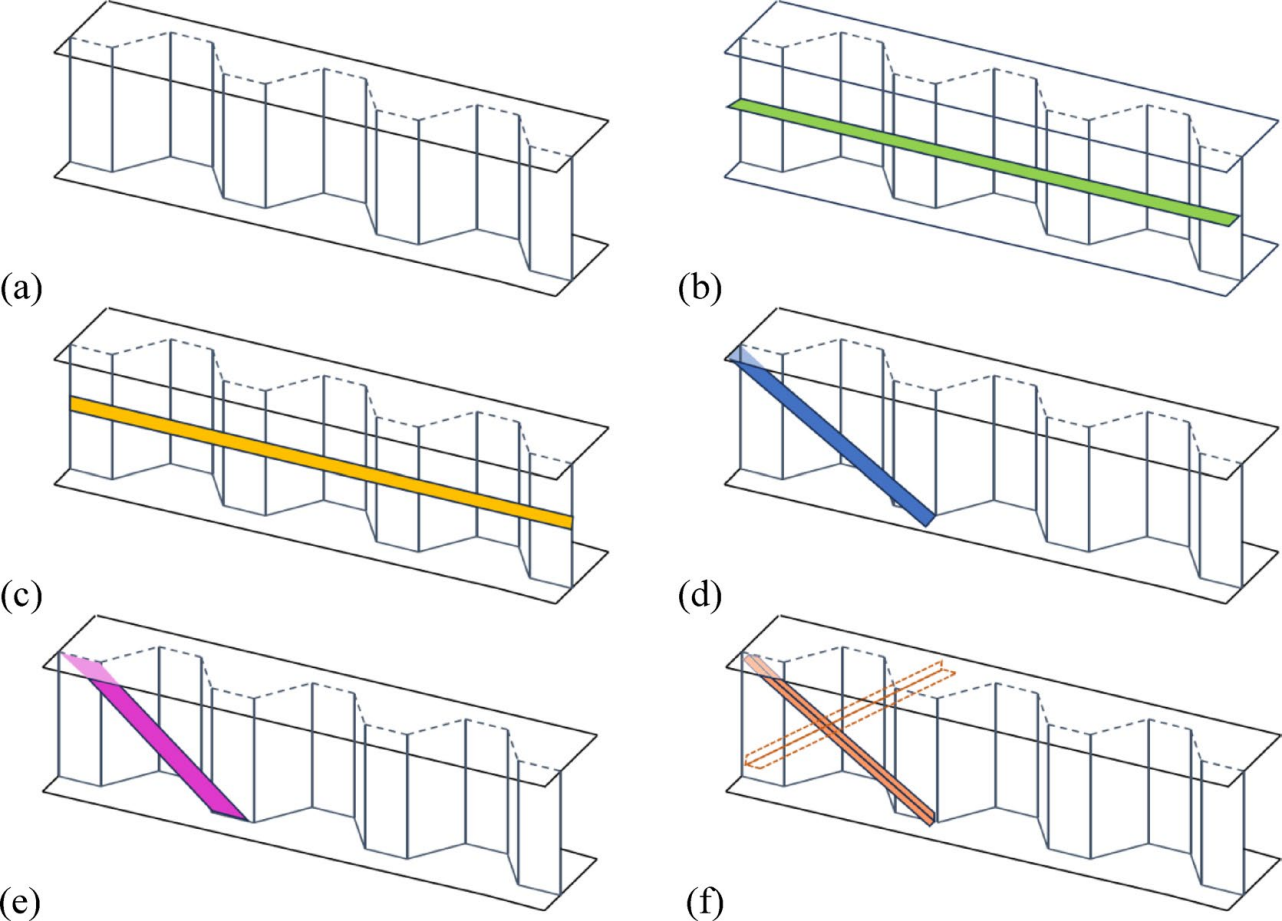
CWBs with various stiffener profiles: (**a**) without any stiffener (**b**) at half height horizontal stiffener (**c**) with vertical doubler plates (**d**) with inclined stiffeners (**e**) with diagonal doubler plates (**f**) with bracing stiffeners.

The parametric study of the FE models focused on examining the effects of five stiffener groups on the capacity of CWSBs. The study utilized a series of finite element models based on the control beam, with detailed geometric dimensions provided in following section. The studied key parameters are stiffener type, location, configuration, side placement, and rotation angles. These parameters were analyzed to understand their influence on the CWSBs capacity, shear stress enhancement, and strength-to-weight ratio. The objective was to identify the optimal configurations to enhance the structural performance of CWSBs. Table 4 provides details on the proposed five stiffener configurations, including stiffener locations, side placement, the thickness $({t}_{st})$ and width $({b}_{st})$ of the stiffeners, as well as their corresponding symbols and shapes. In addition, Fig. 7 shows schematic examples of each type of the proposed stiffener configurations.

**Table 4:** Details of five stiffener profiles for different configurations.

Group	Stiff. profile	Config.	Symbol	$t_{st}$ (mm)	$b_{st}$ (mm)	Shape
Control	–	–	B	–	–	
Group1	HZ stiffener	1/5 height	B-Hz-F	6	100	
		1/2 height	B-Hz-C	6	100	
Group 2	Vertical doubler plate	Upper	B-VD-U	4	200	
		Middle	B-VD-M	4	200	
		Lower	B-VD-L	4	200	
Group 3	Inclined stiffeners	Plate	B-I-P	4	100	
		Angle	B-I-A	4	50	
		Box	B-I-Bx	4	50	
Group 4	diagonal doubler plates	1 plate	B-DD-1P	4	200	
		2 plates with spacing	B-DD-2P	4	200	
Group 5	*Bracing stiffeners	Plate	B-Br-P	4	100	
		Angle	B-Br-A	4	50	
		Box	B-Br-Bx	4	50	
U: upper plate L: lower plate M: medium plate F: 1/5 web height C: 1/2 web height			1P: one plate P: plate stiffener Bx: box stiffener A: angle stiffener I: inclined stiffener Br: bracing stiffener			B: corrugated web beam Hz: horizontal stiffener VD: vertical doubler plate DD: diagonal doubler plate 2P: two plates with spacing *: one in the front and one in the back

#### Control beam dimensions

**Fig. 8:**
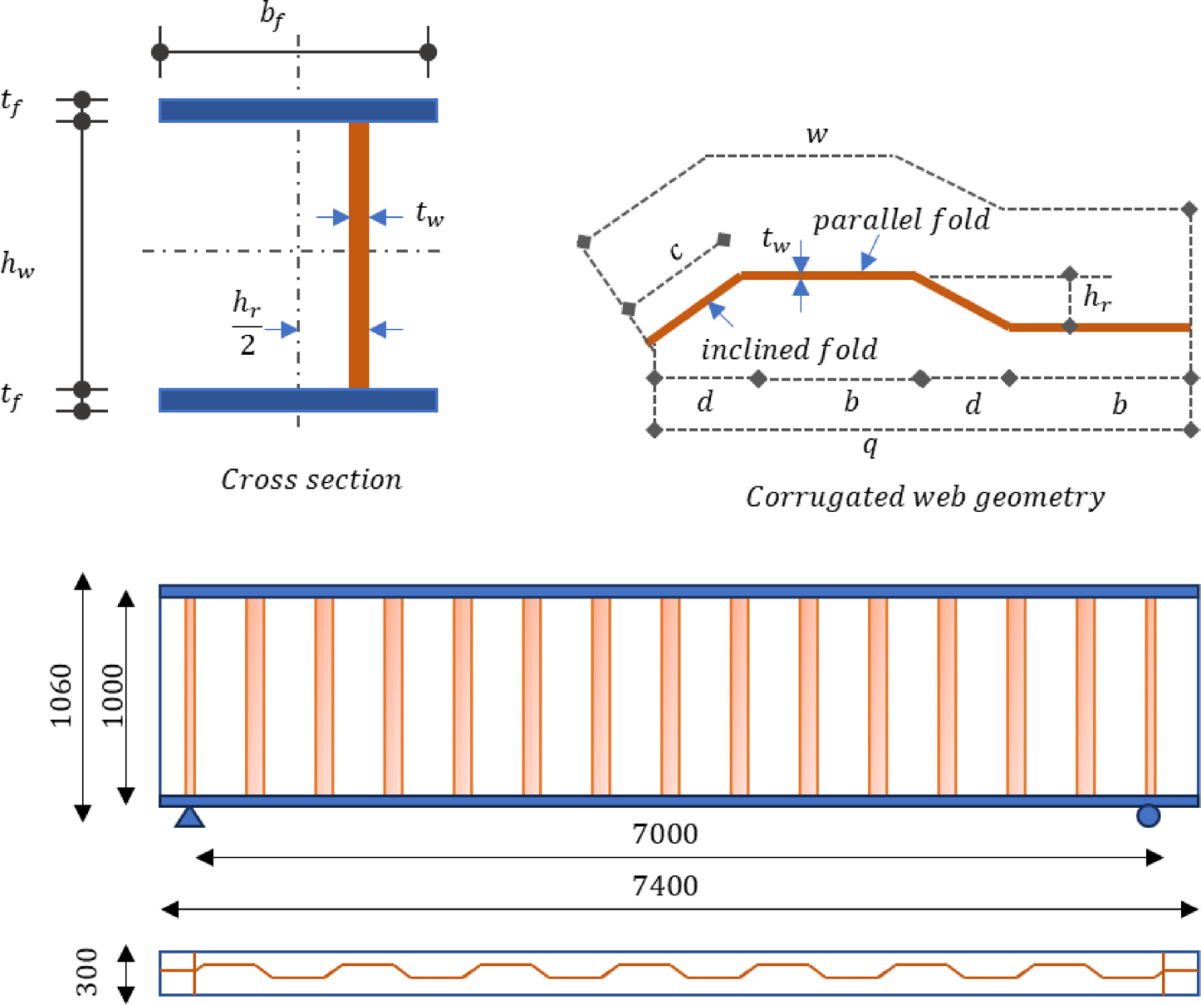
Layout and dimension of control CWSBs.

Several models were prepared to investigate the effect of different stiffener configurations and arrangement on beam capacity. The numerical model used in the current study consists of a simply supported beam subjected to uniform load as shown in Fig. 8. The panel and corrugation profile dimensions are as follows: the unit wavelength of corrugated web ($w$) is 760 mm, horizontal length of one corrugation (q) is 700 mm, parallel fold $\left(b\right)$, inclined fold width $\left(c\right)$, and the projected width of inclined fold $(d)$ is 200 mm, 180.3 mm, and 150 mm respectively. The corrugation depth ($hr$) and angle are 100 mm and 34°, respectively. Span of simple beam ($S$) between supports was 7 m which consisted of 10 full corrugation waves. The beam had a total length ($L$) of 7400 mm. The web height $\left({h}_{w}\right)$ and thickness (${t}_{w}$) were 1000 mm and 4 mm, respectively. The top and bottom flanges were 300 mm wide and 30 mm thick to provide sufficient bending stiffness and to ensure that the failure modes of the test specimens are limited to shear buckling or yielding of the steel web. Vertical stiffeners with a thickness of 25 mm, width of 150 mm and height of 1000 mm were welded at end supports to prevent flange buckling or local buckling of the web.

#### CWSBs with horizontal stiffener

The first group investigates the effect of horizontal stiffeners on CWSBs, four configurations were analyzed to understand the impact of stiffener position and side placement on beam performance. The horizontal stiffener was welded with corrugated steel web at 1/5th of the web’s height (200 mm from the top flange) and at the center of the web on one side and both sides. Details for different configurations of this group are shown in Table 4. One of the four configurations is illustrated in Fig. 7b.

#### CWSBs with vertical doubler plates

The second group focuses on vertical doubler plates attached to web to strengthen corrugated web beams; the effects of plate positioning and side placement were examined. This group is divided into six configurations with doubler plates placed at the top, center, and bottom of the web, on either one or both sides. Table 4 presents the details for the various configurations of this group, while one of the six configurations is illustrated in Fig. 7c.

#### CWSBs with inclined stiffeners

The third group examines CWSBs strengthened by various inclined stiffeners. These inclined stiffeners are positioned close to the left and right supports of the beam. Three types of stiffeners were tested: inclined angle, box and plate stiffeners. The configurations for this group are detailed in Table 4. These stiffeners were placed at angles of 45°, 50° and 60°, with variations in placement (one or both sides of the web). One of these configurations is shown in Fig. 7d.

#### CWSBs with diagonal doubler plates

The fourth group analyzes the use of diagonal doubler plates inclined at angles of 45°, 50° and 60° to enhance the performance of CWSBs. These diagonal doubler plates are positioned close to the left and right supports of the beam. The tested configurations include two arrangements: one doubler plate or two doubler plates with spacing 700 mm. Each of the above-mentioned arrangements has two placements: one side or both sides of the web. Table 4 presents the details for the various configurations of this group. An example of one configuration is shown in Fig. 7e.

#### CWSBs with bracing stiffeners

The fifth group examines beam with corrugated webs enhanced by bracing stiffeners, including bracing angle, box and plate stiffeners. These bracing stiffeners are positioned close to the left and right supports of the beam. Details for different configurations of this group are shown in Table 4. These stiffeners were applied at angles of 45°, 50°, and 60°. One of these configurations was represented in Fig. 7f. All the symbols of the investigated beams were stated in Table 4 in which, each symbol indicates the stiffener profile and configuration. For example, the symbol B-Hz-F specifies a corrugated web beam with horizontal stiffener positioned at 1/5 height of the beam’s web and placed at 1 or 2 sides of the web. While, the symbol B-Br-A specifies a corrugated web beam with bracing stiffener with an angle configuration placed at 1or 2 sides of the web.

## Results and discussions

**Fig. 9:**
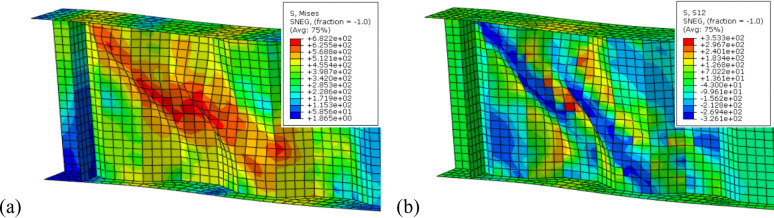
CWSBs without stiffener (reference beam) (**a**) failure mechanism, (**b**) shear stress distribution.

This section discusses the results of different strengthening profiles analyzed using FEM. The results illustrate the effect of the proposed profiles on enhancing the structural load capacity and shear strength of CWSBs compared to the control beam. Moreover, the analysis aims to evaluate strength-to-weight ratio and identify the optimum configuration to enhance the structural performance of CWSBs. In the following subsections, the effect of each profile based on the stiffening configuration of CWSBs are discussed in detail. Table 5 includes stiffener geometric details of investigated models, load-carrying capacity (${P}_{ult}$), mid span deflection (Δ), enhancement ratio in load carrying-capacity, reduction ratio in shear stress, weight of added stiffener, strength-to-weight ratio of added stiffener and the shear stress (${\tau }_{233}$) which resulted from applying the ultimate load of control beam to each of the investigated models. The control beam without stiffeners had an ultimate load of 233 tons and a deflection of 17 mm. Figure 9a shows that control beam failed due to web buckling in the regions involving folds 2–4 near the support where shear stress distribution was high as shown in Fig. 9b. Therefore, strengthening elements were placed in these regions as stated below.

**Table 5:** Results and Performance Comparison of Strengthened CWSBs.

Group	Stiffener. profile	Config.	Sides	inclination angles	Spacing	${P}_{ult}$ (ton)	$\Delta$ (mm)	% Enhancement in load-capacity	${\tau }_{233}$ (MPa)	%Reduction in shear stress	Wt. of stiffener (kg)	Strength to weight ratio (ton/kg)
Control		–	–	–	–	233.20	16.9	–-	265.82			
Group 1	HZ stiffener	1/5 height	1 Side	–	–	259.35	18.8	10.16	248.31	7.05	21.98	1.20
			2 Sides	–	–	259.50	18.75	10.21	248.60	6.93	43.96	0.60
		1/2 height	1 Side	–	–	236.74	17.11	1.58	259.34	2.50	21.98	0.17
			2 Sides	–	–	245.06	18.02	4.92	252.80	5.15	43.96	0.27
Group 2	Vertical doubler plates	Upper	1 Side	–	–	258.62	18.63	9.91	261.62	1.61	43.96	0.58
			2 Sides	–	–	262.12	18.45	11.11	258.87	2.68	87.92	0.33
		Middle	1 Side	–	–	264.54	19.1	11.92	241.29	10.17	43.96	0.73
			2 Sides	–	–	278.69	20.91	16.39	236.61	12.34	87.92	0.52
		Lower	1 Side	–	–	253.83	17.91	8.21	257.80	3.11	43.96	0.47
			2 Sides	–	–	258.01	17.71	9.69	254.52	4.44	87.92	0.28
Group 3	Inclined stiffener	Box	1 Side	45°	–	264.88	18.92	12.04	227.70	16.74	7.92	4.04
				50°	–	254.65	18.10	8.50	241.47	10.08	8.67	2.51
				60°	–	244.30	17.39	4.63	246.38	7.89	11.05	1.02
			2 Sides	45°	–	298.27	21.93	21.88	205.54	29.96	15.83	4.12
				50°	–	279.81	22.00	16.73	206.62	28.65	17.33	2.70
				60°	–	266.52	18.92	12.58	215.13	23.56	22.11	1.53
		Angle	1 Side	45°	–	249.62	17.79	6.66	231.27	14.94	4.26	3.90
				50°	–	247.02	17.38	5.68	249.21	6.66	4.69	2.99
				60°	–	242.67	17.69	3.98	261.22	1.76	6.03	1.60
			2 Sides	45°	–	274.18	19.43	15.02	218.29	21.78	8.52	4.83
				50°	–	261.06	18.64	10.75	235.30	12.97	9.37	2.99
				60°	–	254.34	17.59	8.39	256.32	3.71	12.06	1.77
		Plate	1 Side	45°	–	247.62	17.73	5.90	247.34	7.47	4.55	3.21
				50°	–	245.10	17.50	4.94	250.90	5.95	5.40	2.24
				60°	–	242.66	17.32	3.98	252.19	5.41	6.33	1.53
			2 Sides	45°	–	270.95	19.12	14.01	227.68	16.75	9.11	4.17
				50°	–	261.98	18.42	11.06	241.78	9.94	10.80	2.68
				60°	–	255.56	17.94	8.83	248.37	7.03	12.66	1.78
Group 4	Diagonal doubler plate	Plate	1 Side	45°	–	241.34	17.14	3.46	259.00	2.63	8.88	0.94
					700	249.8	17.15	6.73	250.33	6.19	17.76	0.95
			2Sides		–	265.97	18.80	12.40	230.00	15.57	17.76	1.86
					700	269.21	18.47	13.45	220.19	20.72	35.52	1.02
			1 Side	50°	–	235.00	16.49	0.85	262.63	1.22	9.77	0.21
					700	242.90	16.64	4.08	260.98	1.85	19.53	0.51
			2Sides		–	252.90	17.90	7.87	234.23	13.49	19.53	1.02
					700	259.34	18.20	10.16	226.14	17.55	39.06	0.67
			1 Side	60°	–	233.00	16.16	0.13	265.70	0.05	12.56	0.02
					700	239.44	16.95	2.69	264.73	0.41	25.12	0.26
			2Sides		–	235.10	16.45	0.89	259.78	2.33	25.12	0.08
					700	241.02	16.47	3.33	250.29	6.21	50.24	0.16
Group 5	Bracing stiffener	Box		45°	–	312.30	21.82	25.39	205.97	29.06	15.83	5.01
				50°		291.37	20.26	20.03	207.87	27.88	17.33	3.37
				60°		270.99	18.81	14.02	228.96	16.10	22.11	1.72
		Angle		45°	–	280.06	19.75	16.80	230.49	15.33	8.52	5.52
				50°		273.28	19.74	14.74	232.91	14.13	9.37	4.30
				60°		268.7	19.57	13.29	234.34	13.43	12.06	2.96
		Plate		45°	–	277.33	19.76	16.00	233.52	13.83	9.11	4.87
				50°		274.72	19.71	15.19	235.74	12.76	10.80	3.86
				60°		271.20	19.43	14.09	238.27	11.56	12.66	3.02

### CWSBs with horizontal stiffeners

In the first group, the aim is to evaluate how horizontal stiffeners can enhance the performance of CWSBs including the enhancement of the ultimate load capacity and shear stress distribution of the beams. Four models were conducted to investigate different configurations: a horizontal stiffener placed at 1/5th of the web height (B-Hz-F) from one side and from both sides and a horizontal stiffener positioned at the center of the web (B-Hz-C) from one side and from both sides. The results of this group are presented in Table 5 and explained in the following paragraph.

**Fig. 10:**
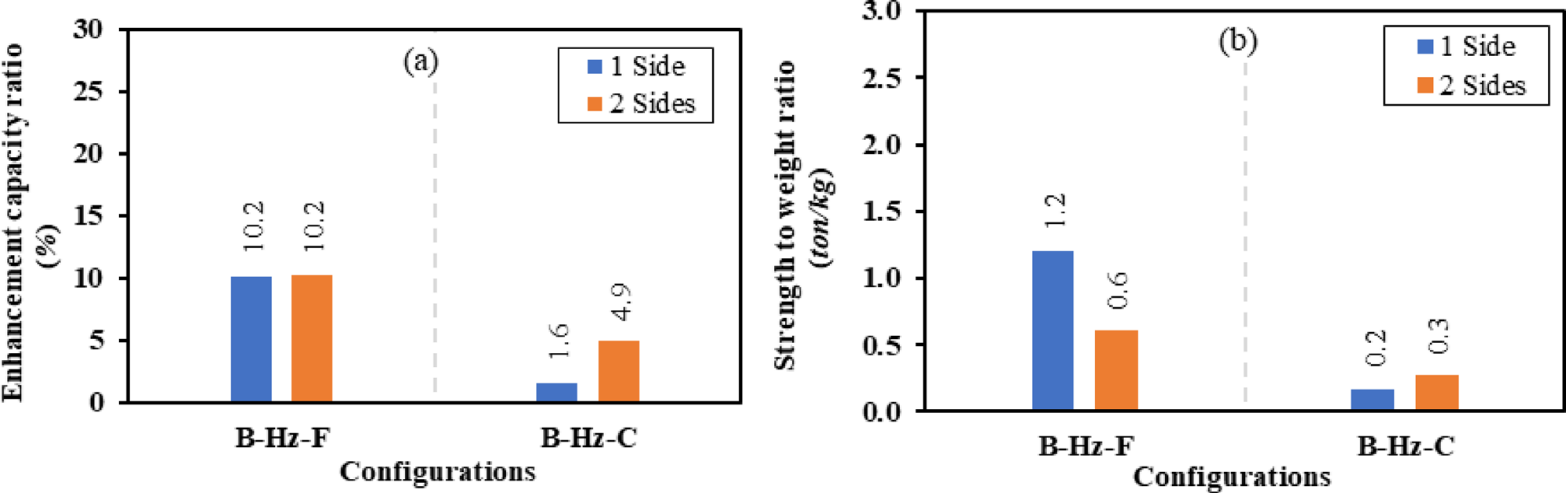
(**a**) Enhancement ratio of load-carrying capacity; (**b**) Strength-to-weight ratio for CWSBs with horizontal stiffeners.

Figure 10a shows the effect of using different configurations of horizontal stiffeners on the enhancement ratio of ultimate load capacity of CWSBs. The ultimate load improvement ratio of (B-Hz-F) on one side or both sides of the web increased to 10.16% and 10.21% with shear stress reduction represented 7.05% and 6.93%, respectively as indicated in Table 5. The results indicate that placing stiffeners on both sides caused the enhancement of load-capacity to remain approximately constant when compared to a single-side stiffener, but the strength-to-weight ratio decreased due to the increased weight of the stiffener as illustrated in Fig. 10b. On the other hand, (B-Hz-C) did not perform as well as (B-Hz-F). When using the horizontal stiffener at center of web’s height placed at one and two sides, the improvement ratio of load carrying capacity was increased by 1.58% and 4.92% and shear stress was reduced by 2.50% and 5.15% respectively. The deflection of profile remained close to the control beam’s values.

**Fig. 11:**
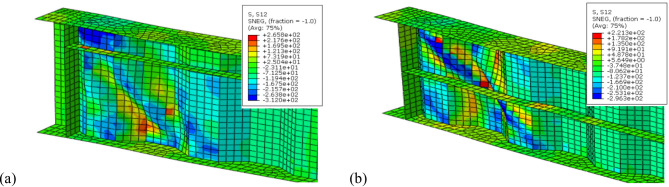
Impact of using horizontal stiffener with on the CWSBs failure mode (**a**) at fifth web height on one side, (**b**) at the center of the web on one side.

In summary, a horizontal stiffener placed at 1/5th of the web height (B-Hz-F) is more efficient at enhancing the performance of CWSBs compared to central stiffeners (B-Hz-C), as the stiffeners are located close to the compression flange, besides, they provide more efficient shear stress distribution and contribute to improved ultimate load capacity. Moreover, using (B-Hz-F) effectively restrains the horizontal part of the fold near the upper part of the web where the failure occurred, as shown in Fig. 11a. In contrast, placing stiffeners at mid-height (B-Hz-C) does not adequately restrain the inclined fold where the failure occurred, as shown in Fig. 11b.

### CWSBs with vertical doubler plate

**Fig. 12:**
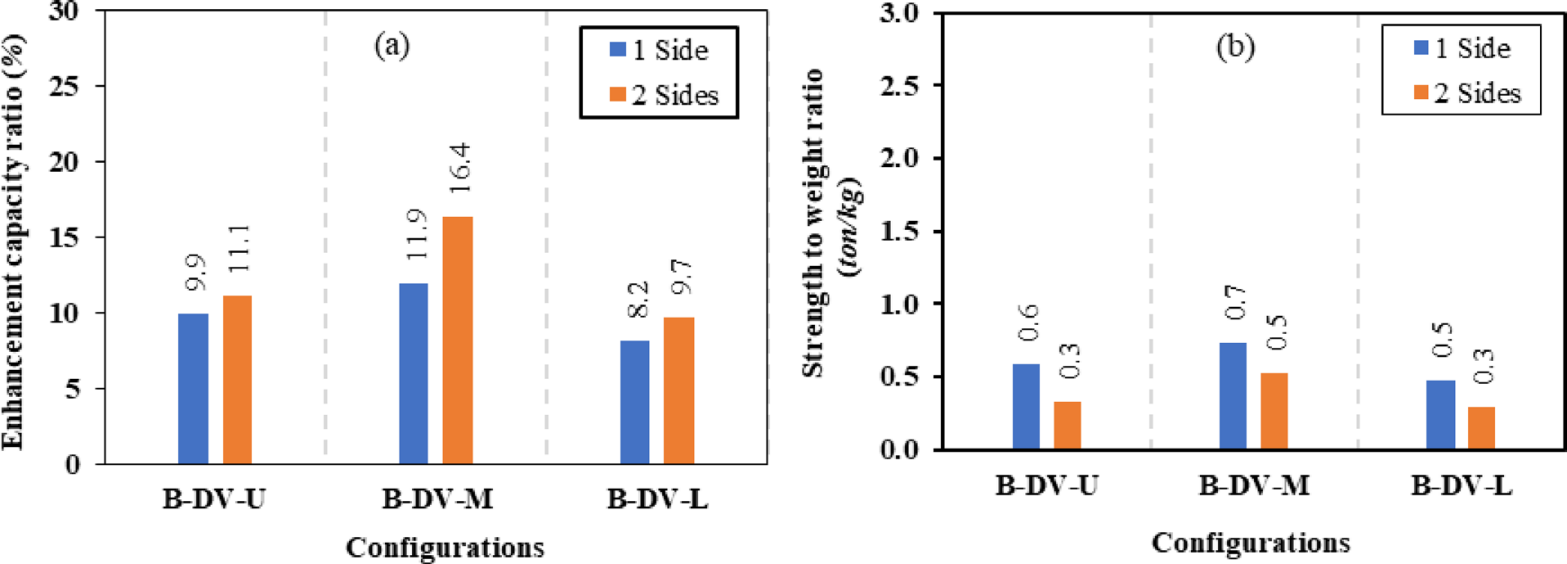
Load versus deflection curve for CWSBs with horizontal stiffener from (**a**) one side (**b**) two sides.

The second group of models is divided into six configurations examining CWSBs reinforced by vertical doubler plates, investigating the effects of plate’s placement (upper, middle, and lower) and whether attached on one or both sides of CWSBs on shear resistance and ultimate load capacity. Vertical doubler plates are used to improve the performance of CWSBs by preventing buckling and redistribution of loads. Table 5 and Fig. 12 show the effect of using different configurations of vertical doubler plates on the ultimate load capacity of CWSBs as well as the corresponding strength-to-weight ratio.

For CWSBs with top vertical doubler plates (B-VD-U) at one side, ultimate load improvement ratio was increased by 9.91%, with a slight reduction in shear stress by 1.61%. When plates were applied on both sides, the improvement ratio of ultimate load was improved by 11.11% and the shear stress was reduced by 2.68%.

Using CWSBs with plates at the center (B-VD-M) on one side increased the enhancement ratio of ultimate load by 11.92% and a shear stress reduction of 10.17%. When plates were applied to both sides, the ultimate load reached an improvement ratio of 16.39%, and a reduction ratio of 12.34% in shear stress. Finally, enhancement ratio of CWSBs with plates at the bottom (B-VD-L) on one side, increased the ultimate load by 8.21%, with a relatively low reduction in shear stress 3.11%. When using bottom plates on both sides, the ultimate load improved by 9.69%, with a 4.44% reduction in shear stress, but decreased the strength-to-weight ratio when compared to bottom plates on one side.

**Fig. 13:**
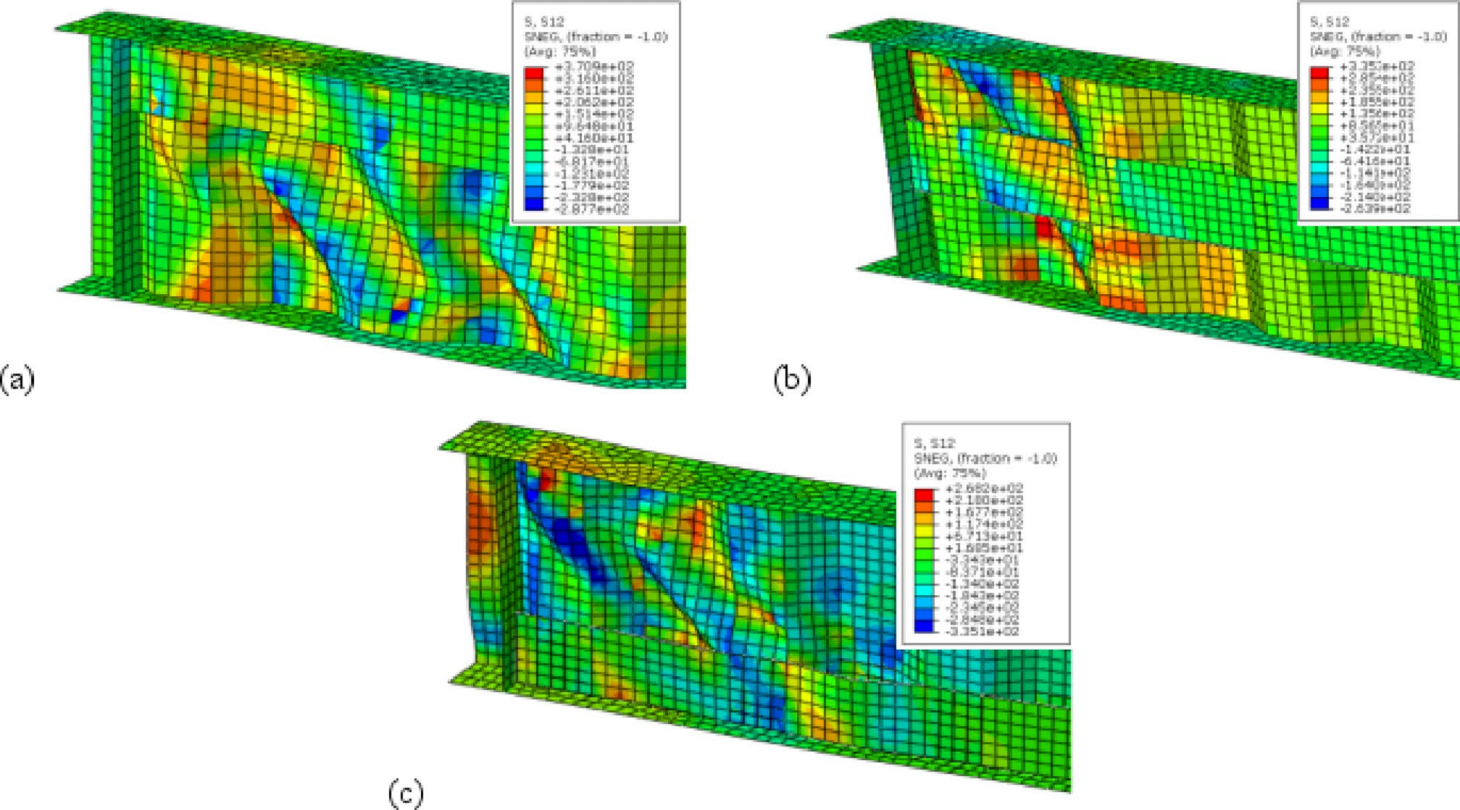
Failure shape of CWSBs with vertical doubler plate (**a**) at top web, (**b**) at center web, (**c**) at the bottom web.

In conclusion, placing plates at middle height of the web (B-VD-M), especially in two-sided configurations, provides the most significant improvements in ultimate load capacity and shear strength but the strength-to-weight ratio slightly decreases compared to one-sided placement. This is because middle doubler plates effectively divide the web into two equal parts, reducing the buckling length Fig. 13b and enhancing shear buckling resistance compared to upper (B-VD-U) or lower plates (B-VD-L) in which the buckling length (defined as the web height minus the plate height) is higher as shown Fig. 13a and c. Additionally, middle plates are better than the upper plates while the lower plates come in the third place as strengthening methods in this group.

### CWSBs with inclined stiffeners

In the third group of models, CWSBs were enhanced using various inclined stiffeners in three configurations, angle, box, and plate stiffeners, applied at varying inclination angles 45°, 50°, and 60°. These stiffeners were tested on one side and both sides of the web to evaluate their effects on the ultimate load capacity, strength-to-weight ratio and shear stress of CWSBs compared to control beam. The results of this group are shown in Table 5.

Firstly, for inclination angle of 45°, the inclined box stiffeners (B-I-Bx) on one side and two sides achieved a 12.04% and 21.88% improvement ratio in ultimate load, which corresponds to a 16.74% and 29.96% reduction ratio in shear stress, respectively. While for the inclined angle stiffeners (B-I-A) on one side and two sides exhibit enhancements ratio in load capacity of 6.66% and 15.02% corresponding to 14.94% and 21.78% reduction in shear stress, respectively. Also, for the inclined plate stiffeners (B-I-P) positioned on one side and two sides showed a rise in ultimate load capacity of 5.90% and 14.01% with shear stress decrease of 7.47% and 16.75%, respectively.

Secondly, the inclined box stiffeners (B-I-Bx) at 50° on one side and two sides showed 8.50% and 16.73% increase in ultimate load corresponding to a 10.08% and 28.65% reduction in shear stress, respectively. While for the inclined angle stiffeners (B-I-A) on one side and two sides exhibit improvement in load capacity of 5.68% and 10.75% corresponding to 6.66% and 12.97% decline in shear stress, respectively. Also, for the inclined plate stiffeners (B-I-P) positioned on one side and two sides showed improvement in ultimate load capacity of 4.94% and 11.06% with shear stress decline of 5.95% and 9.94%, respectively.

Finally, the inclined box stiffeners (B-I-Bx) at 60° on one side and two sides attained a 4.63% and 12.58% development in ultimate load corresponding to 7.89% and 23.56% reduction in shear stress, respectively. While for the inclined angle stiffeners (B-I-A) on one side and two sides exhibit an increase in load capacity of 3.99% and 8.39% corresponding to 1.76% and 3.71% decrease in shear stress, respectively. Also, for the inclined plate stiffeners (B-I-P) positioned on one side and two sides showed improvement in ultimate load capacity of 3.98% and 8.83% while the shear stress was lowered by a ratio of 5.41% and 7.03%, respectively.

**Fig. 14:**
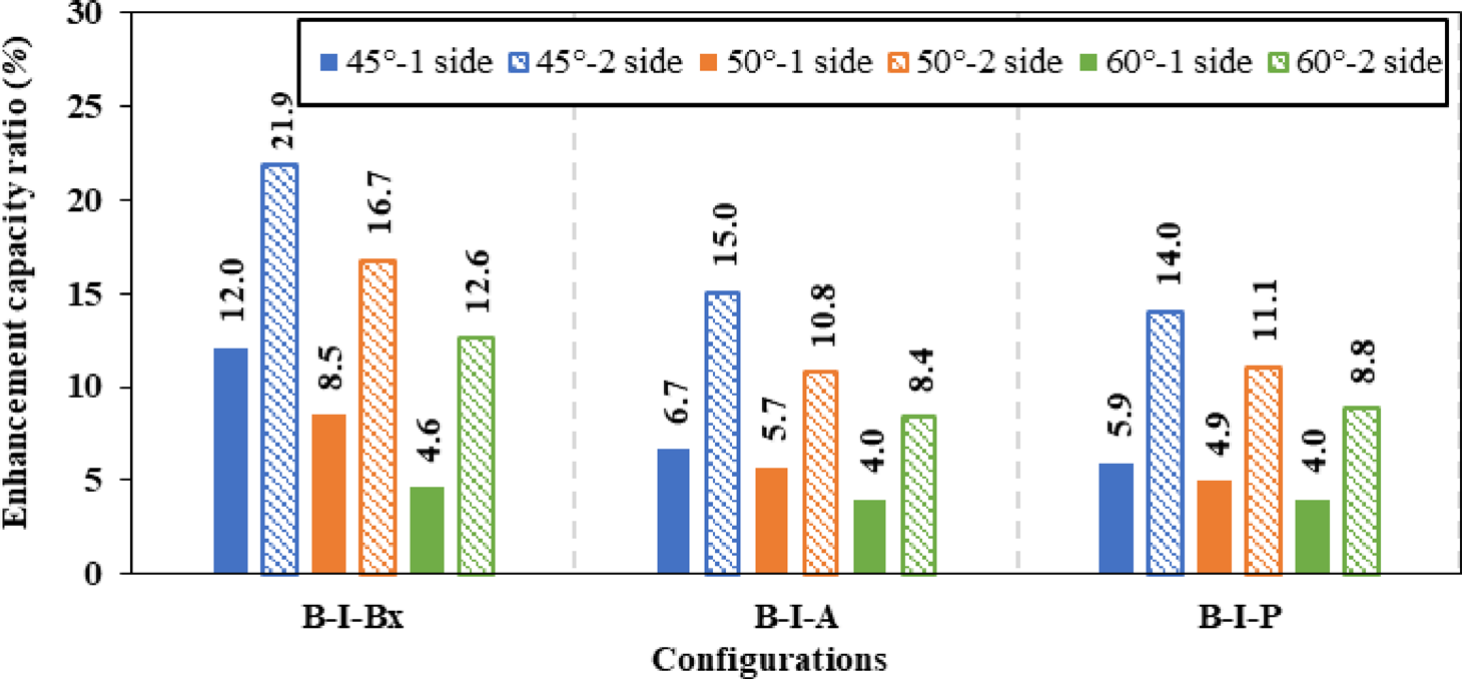
Enhancement ratio of the load-carrying capacity of CWSBs with inclined stiffeners.

**Fig. 15:**
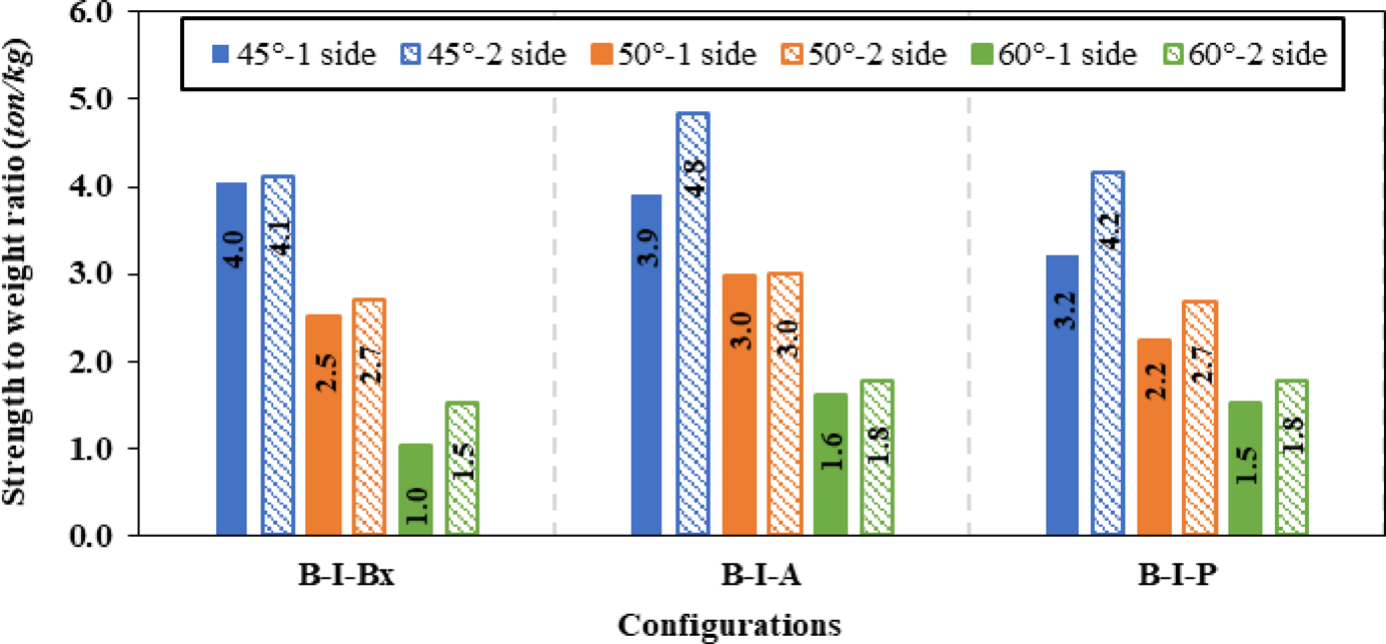
Strength-to-weight ratio of CWSBs with inclined stiffeners.

Figure 14 illustrates the effect of varying the inclination angles of different inclined stiffeners (box, angle, and plate) on the enhancement’s ratio of ultimate load carrying capacity. Figure 15 presents the corresponding variations in the strength-to-weight ratio of (CWSBs) with respect to the same stiffener configurations and inclination angles. The analysis compares one-sided and double-sided stiffener configurations.

**Fig. 16:**
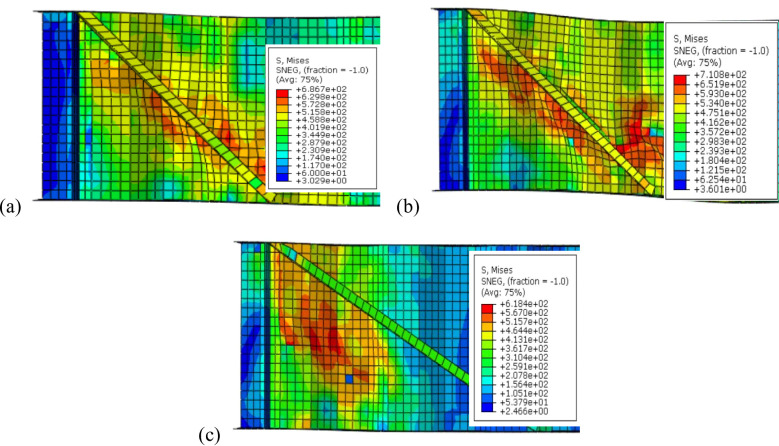
Influence of box stiffener inclination on failure locations of the CWSBs (**a**) 45°, (**b**) 50°, (**c**) 60°.

To sum up, the obtained results indicate that, optimal angle for three configurations is 45° as it is parallel to the failure mode Fig. 16a while increasing the inclination angle to 50° or 60° results in lower improvement ratios for ultimate load as the stiffeners deviate from the stress direction, as shown in Fig. 16b and c.

Strength-to-weight ratio was used to determine the optimum stiffener number of sides for a fixed inclination angle of 45°. For (B-I-Bx) on one side and two sides, strength-to-weight ratio reached 4.03 and 4.12, respectively. While for (B-I-A) on one side and two sides and (B-I-P) on one side and two sides values of 3.90, 4.83, 3.21 and 4.17 respectively were achieved. These results show that two-sided stiffeners are better than one-sided stiffeners at constant inclination angle (45°). This is because the two-sided inclined stiffener restrains the horizontal fold from both sides, whereas the inclined stiffener on one side only restrains the horizontal fold from one side and allow buckling for the other side.

To choose the superior stiffener configuration (Box, Angle or Plate), a certain type of inclined stiffener was chosen (two sided with 45° inclination angle). Box, angle and plate stiffeners achieved strength-to-weight ratios of 4.12, 4.83, and 4.17 respectively. These results suggest that the best inclined stiffener configuration is the angle stiffener (B-I-A). As the angle provides stiffening to the web using less steel area compared to box stiffener and in the same time the angle provides more stiffness and inertia compared to the plate stiffeners.

### CWSBs with diagonal doubler plates

In the fourth group of models, beams with corrugated webs were enhanced using various diagonal doubler plates applied at various angles 45°, 50°, and 60° from one side and two sides of the beam’s web. These diagonal doubler plates are positioned close to the left and right supports of the beam. The diagonal doubler plate includes two arrangements: the first involves attaching one diagonal doubler plate to the beam’s web while the other arrangement involves utilizing two diagonal doubler plate with spacing 700 mm. The diagonal doubler plates displayed a varying performance based on their angle, number of sides and arrangement. The results for this group are shown in Table 5.

Firstly, diagonal doubler plate at 45° inclination angle on one side and both sides reached a ratio of 3.46% and 12.40% improvement in ultimate load with 2.63% and 15.57% reduction in shear stress, respectively. While for the other arrangement (spacing of 700 mm), the ultimate load improvement ratio increased to 6.73% and 13.45% with corresponding reduction in shear stress of 6.19% and 20.72% for one sided and both sided plates respectively. The same comparison method was applied for the diagonal doubler plate inclined at 50° and 60o with the corresponding results shown in Table 5.

**Fig. 17:**
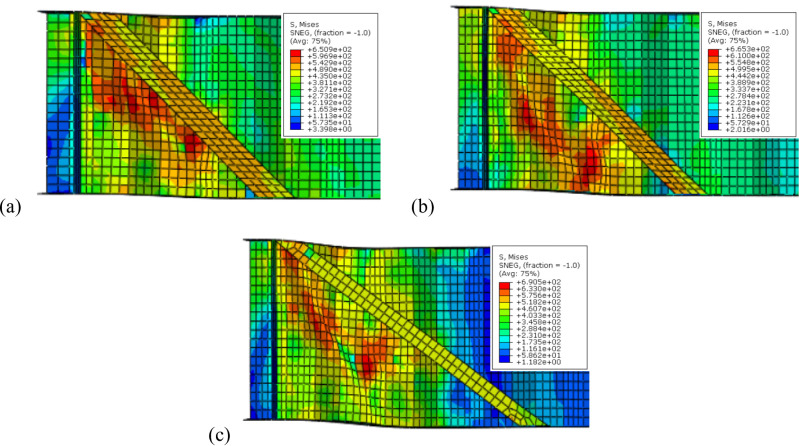
Failure locations of CWSBs with diagonal doubler plates (first arrangement) at inclination angles (**a**) 45°, (**b**) 50°, and (**c**) 60°.

Based on the previous data, it is worth mentioning that 45° is the most effective inclination angle for diagonal doubler plates in the two arrangements (one plate or two plates spaced 700 mm) as it is parallel to the failure mode Fig. 17. Increasing the angle to 50° or 60° results in declination of improvement ratio of ultimate load capacity as the stiffeners deviate from the failure mode.

**Fig. 18:**
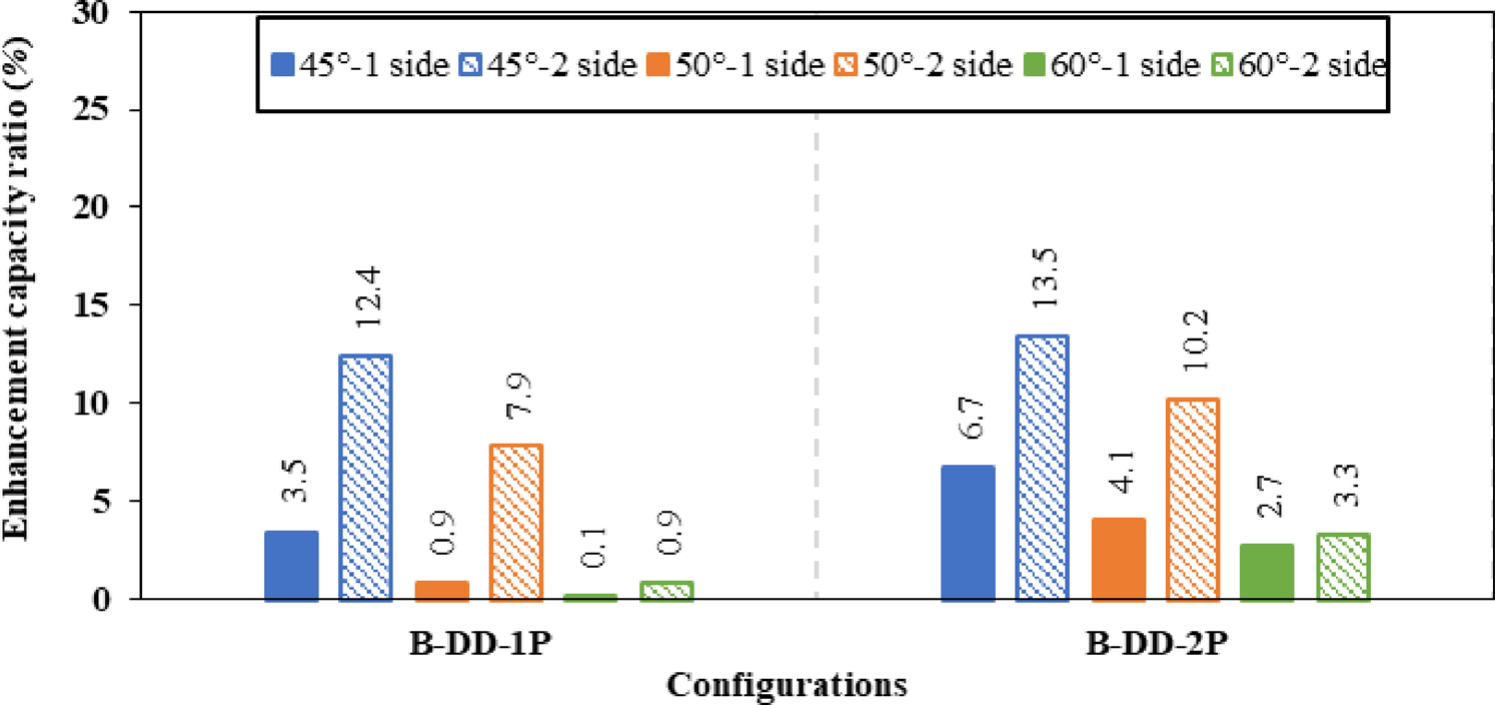
Enhancement ratio of the load-carrying capacity of CWSBs with diagonal doubler plates.

**Fig. 19:**
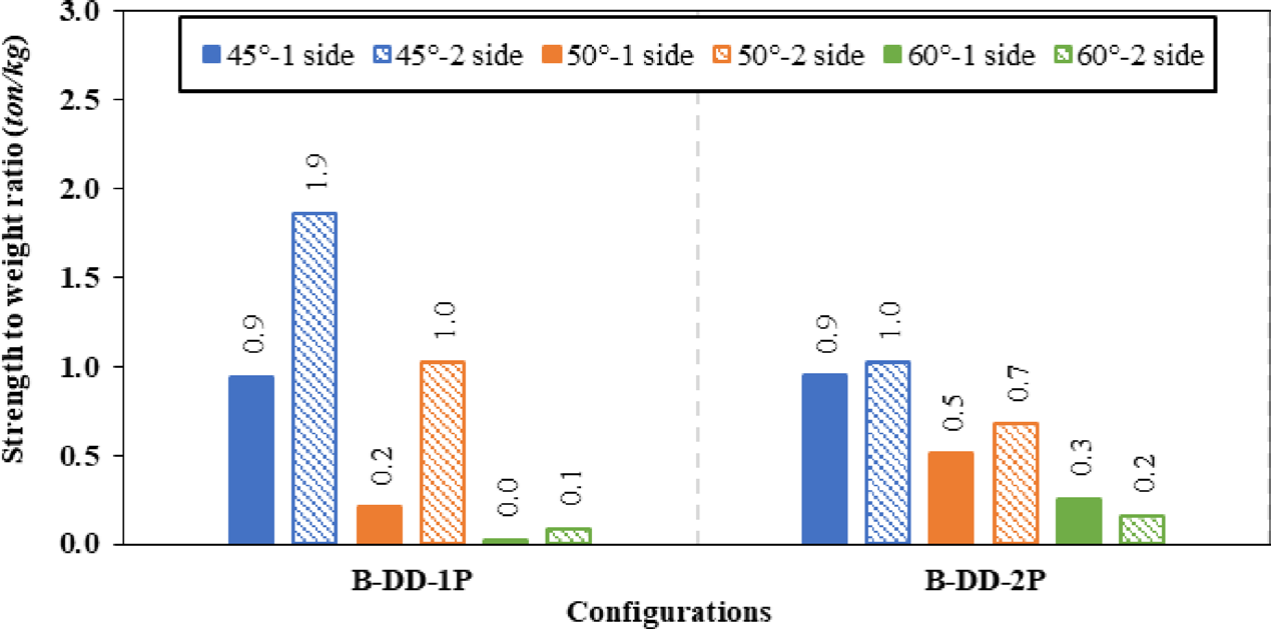
Strength-to-weight ratio of CWSBs with diagonal doubler plates.

The strength-to-weight ratio was utilized to evaluate the effect of the number of sides of diagonal doubler plates at a fixed 45° inclination angle which is identified as the most effective angle. Results indicated that using diagonal doubler plates on both sides of the web significantly enhances the strength-to-weight ratio compared to one-sided diagonal doubler plates. Specifically, the two-sided first arrangement (without spacing) achieved the highest ratio of 1.86 reflecting superior structural efficiency. Although the two-sided second arrangement reached 1.02, it remained considerably less efficient than the two-sided first arrangement. Therefore, the optimal arrangement is the two-sided diagonal doubler plate without spacing at a 45° inclination, as it provides the most effective enhancement in load capacity relative to the added weight. Figures 18, and 19 illustrate the impact of varying the inclination angles (45°, 50° and 60°), number of sides and arrangement of diagonal doubler plate on the ultimate load capacity and strength-to-weight ratio of (CWSBs).

### CWSBs with bracing stiffeners

**Fig. 20:**
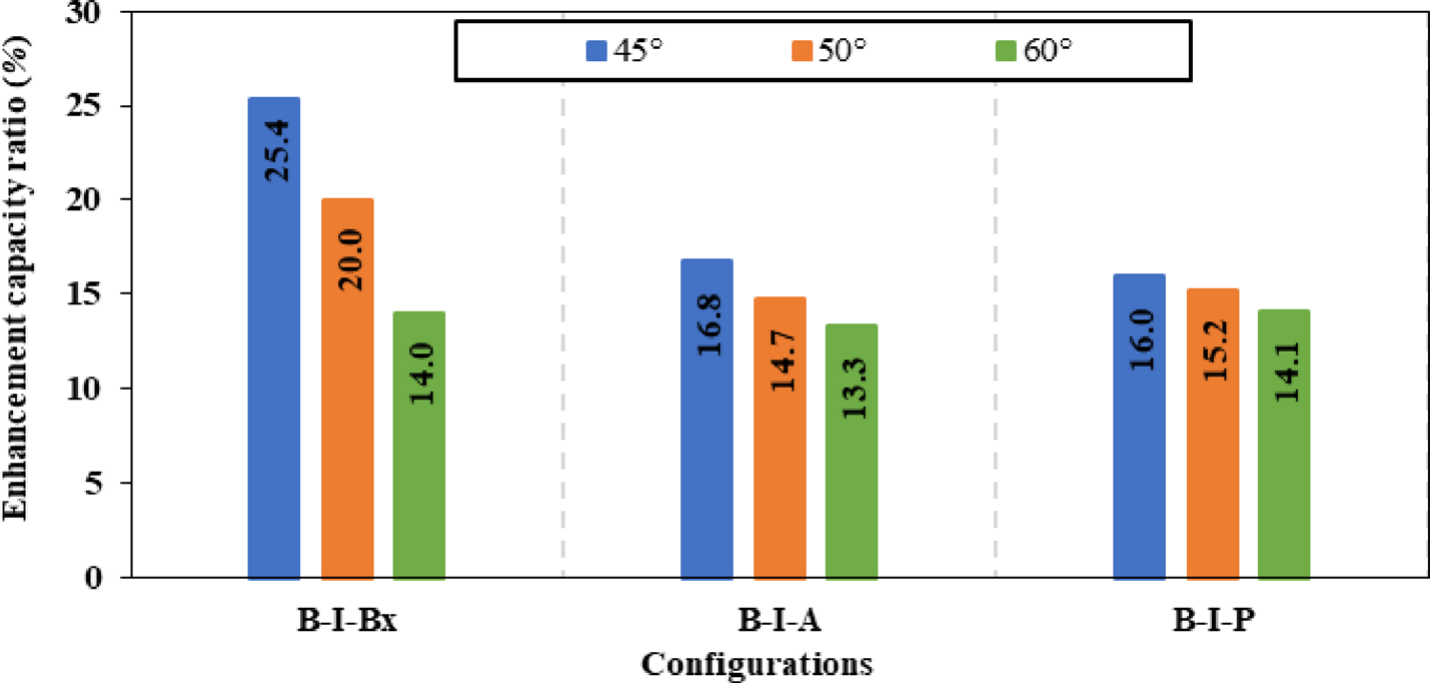
Enhancement ratio of the load-carrying capacity of CWSBs with bracing stiffeners.

**Fig. 21:**
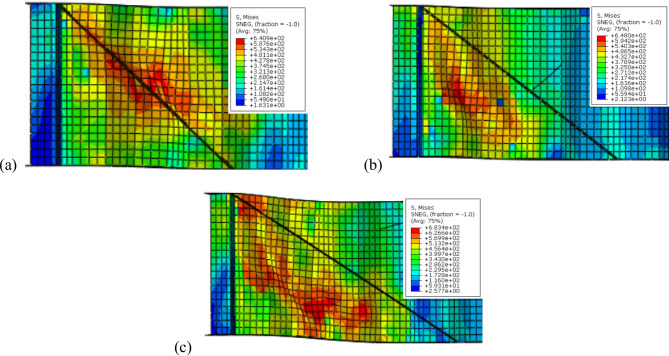
Location of failure for CWSBs via bracing plate stiffeners (**a**) 45°, (**b**) 50°, (**c**) 60° inclination angles.

In the fifth group of models for CWSBs different configurations for bracing stiffeners were used, including bracing angle, box and Plate stiffeners, placed at various angles 45°, 50°, and 60°. Table 5 presents the results of this group. The key factors that were considered to choose the optimum inclination angle for different configuration of bracing stiffeners are the improvement ratio of ultimate load and the reduction of shear strength. The angle bracing stiffeners at 45° inclination angle achieved a ratio of 16.80% improvement in ultimate load, which corresponded to 15.33% reduction in shear stress. While for the box bracing stiffeners, an enhancement ratio of 25.39% was obtained in load capacity with a corresponding ratio of 29.06% reduction in shear stress. In addition, for the bracing plate stiffener, improvement ratio of 16.00% was reached in load-capacity, and a decline of 13.83% in shear stress. The same procedure was applied for 50° and 60° inclination angles for all the utilized bracing stiffener configurations (angle, box and plate). The results show that the inclination angle of 45° provided the highest load-capacity as indicated in Fig. 20. This is because the 45° inclination angle aligns with the failure mode unlike the 50° and 60° inclination angles that diverge from the stress direction as demonstrated in Fig. 21. This proves that increasing the rotation angle reduces the effectiveness of the bracing stiffeners.

**Fig. 22:**
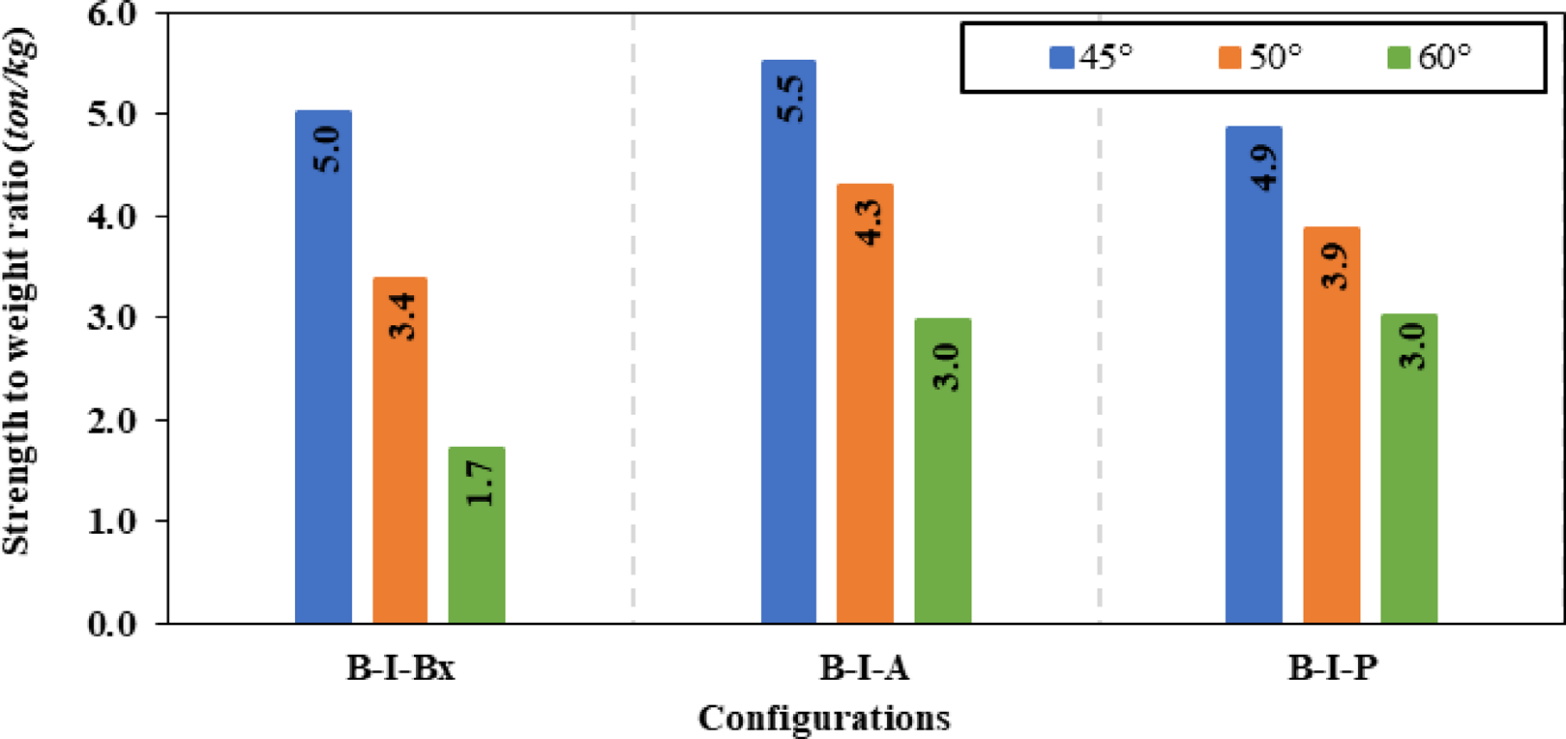
Strength-to-weight ratio of CWSBs with bracing stiffeners.

The value of strength-to-weight ratio was selected to find the optimum configuration for bracing stiffener (angle, box and plate) while the value of inclination angle was kept constant at 45°. Angle, box and Plate bracing stiffener configurations reached 5.52, 5.01 and 4.87 in strength-to-weight ratio respectively as shown in Fig. 22. These results show that bracing angle configuration outperformed box and plate bracing stiffeners. The unique geometry of angle bracing stiffeners allowed them to achieve superior results over the plate or box stiffeners. As the horizontal leg (free leg) of the angle effectively restrains the corrugated web from shear buckling, while the vertical leg (attached to web) increases the effective thickness, thereby reducing shear stress. Although, CWSBs stiffened with box stiffeners surpassed the angle stiffeners in load-capacity, the former had a significant increase in additional weight compared to the latter. Therefore, the box stiffeners achieved lesser strength-to-weight ratio than the angle stiffeners. In summary, the best bracing stiffener configuration is the bracing angle stiffeners inclined at 45°.

### Optimization

In this sub-section, the tested numerical models were used to determine the optimum configuration, location and inclination angle for each stiffening type (horizontal stiffener or vertical doubler plate or diagonal doubler plate or bracing stiffener or inclined stiffener). Selection of the optimum stiffening technique from the numerically studied beams is based on the value of strength-to-weight ratio of the beams. For the horizontal stiffener, choosing a horizontal stiffener at 1/5 of total beam height from compression flange from one side showed better results in strength-to-weight ratio when compared to other configurations of horizontal stiffener. Regarding vertical doubler plate, using a central plate from one side resulted in better strength-to-weight ratio than upper or lower plates. While the diagonal doubler plate, two-sided configuration at 45° without spacing achieved better values in strength-to-weight ratio than the one-sided or spaced configurations. As for the inclined stiffener, the angle configuration provided a better strength-to-weight ratio when applied on both sides at a 45° angle compared to other inclined stiffener configurations. With respect to the bracing stiffener, the angled configuration at 45° achieved a higher strength-to-weight ratio than the other bracing stiffener configurations. To sum up, the bracing stiffener with a 45° inclined angled configuration is identified as the optimum stiffening technique for corrugated web steel beams.

## Conclusions

The research studied the corrugated web steel beams strengthened using various stiffener configurations. Five distinct stiffener profiles were introduced: horizontal stiffeners, vertical doubler plates, inclined stiffeners, diagonal doubler plates, and bracing stiffeners. Evaluation performance was done using finite element model. Comparison between different strengthening types of CWSBs should carefully consider optimizing structural performance while managing material consumption, represented by the strength-to-weight ratio. The following conclusions can be drawn from the obtained results.

- The optimal angle for inclined stiffeners on CWSBs is 45°, as this angle is parallel to the failure mode. Performance declined with increased inclination angles, as the stiffeners deviate from the stress direction. But even at these angles, stiffeners provided enhancements over the control beam.
- Both two-sided horizontal stiffeners and vertical doubler plates positioned at the center of web height improved load capacity compared to their single-sided counterparts. However, the strength-to-weight ratio was lower in two-sided configurations. Which makes single-sided configurations more efficient.
- Two-sided inclined stiffener configurations at the same angle outperform one-sided configurations achieving more strength-to-weight ratio and improvement ratio in ultimate load capacity up to a 4.831 ton/kg and 21.88% respectively.
- The two-sided angle stiffener inclined at 45° is better than inclined box stiffener while the inclined plate stiffener comes in the third place in terms of strength-to-weight ratio (4.83, 4.17 and 4.12 respectively).
- According to strength-to-weight ratio, positioning the horizontal stiffeners at 1/5 of the web height is found to be more effective than using horizontal stiffeners placed at 1/2 web height.
- Vertical doubler plates positioned at the middle of the web is superior than upper or lower vertical doubler plates with strength-to-weight ratio of 0.73 ton/kg.
- Bracing stiffeners are more effective than two-sided inclined stiffeners, as they strengthen a larger area, providing support to a larger number of horizontal folds of the corrugated web and efficiently distributing shear forces across the web.
- The optimum stiffener configuration is bracing stiffeners (group 5) especially the angle bracing stiffener which was the ideal stiffener shape with the best strength-to-weight ratio of 5.52 ton/kg followed by box bracing stiffeners (5.01) then plate bracing stiffeners (4.8).

Future studies should focus on developing accurate design equations and guidelines for strengthened corrugated web steel beams (CWSBs) to improve the safety and efficiency of these structures. A comprehensive parametric study on bracing angle stiffeners, which have been identified as the most effective configuration, is recommended. This study should explore varying their dimensions and rotation angles to optimize performance. Additionally, further research is needed to assess the behavior of strengthened CWSBs under a variety of loading conditions. Specifically, investigations should focus on their ability to enhance load capacity and shear stress resistance, with a comparative analysis against the current study, which primarily evaluated CWSBs under distributed load conditions. These efforts will provide valuable insights for advancing the design and application of strengthened CWSBs in practical engineering scenarios.

## Data Availability

Data supporting the findings of this study are available from the corresponding author upon reasonable request.
